# Explainable deep learning in plant phenotyping

**DOI:** 10.3389/frai.2023.1203546

**Published:** 2023-09-19

**Authors:** Sakib Mostafa, Debajyoti Mondal, Karim Panjvani, Leon Kochian, Ian Stavness

**Affiliations:** ^1^Department of Computer Science, University of Saskatchewan, Saskatoon, SK, Canada; ^2^Global Institute for Food Security, University of Saskatchewan, Saskatoon, SK, Canada

**Keywords:** explainable AI, deep learning, plant phenotyping, data bias, agriculture

## Abstract

The increasing human population and variable weather conditions, due to climate change, pose a threat to the world's food security. To improve global food security, we need to provide breeders with tools to develop crop cultivars that are more resilient to extreme weather conditions and provide growers with tools to more effectively manage biotic and abiotic stresses in their crops. Plant phenotyping, the measurement of a plant's structural and functional characteristics, has the potential to inform, improve and accelerate both breeders' selections and growers' management decisions. To improve the speed, reliability and scale of plant phenotyping procedures, many researchers have adopted deep learning methods to estimate phenotypic information from images of plants and crops. Despite the successful results of these image-based phenotyping studies, the representations learned by deep learning models remain difficult to interpret, understand, and explain. For this reason, deep learning models are still considered to be black boxes. Explainable AI (XAI) is a promising approach for opening the deep learning model's black box and providing plant scientists with image-based phenotypic information that is interpretable and trustworthy. Although various fields of study have adopted XAI to advance their understanding of deep learning models, it has yet to be well-studied in the context of plant phenotyping research. In this review article, we reviewed existing XAI studies in plant shoot phenotyping, as well as related domains, to help plant researchers understand the benefits of XAI and make it easier for them to integrate XAI into their future studies. An elucidation of the representations within a deep learning model can help researchers explain the model's decisions, relate the features detected by the model to the underlying plant physiology, and enhance the trustworthiness of image-based phenotypic information used in food production systems.

## 1. Introduction

The emergence of deep learning has allowed researchers to perform tasks that were previously thought to be impossible. Deep learning is popularly used in the fields of computer-aided diagnosis (Xie et al., 2021), drug discovery (Chen et al., 2018), healthcare (Esteva et al., 2019), law enforcement (Raaijmakers, 2019), autonomous vehicles (Rao and Frtunikj, 2018), robotics (Sünderhauf et al., 2018), and so on. Research (2020) predicted that the global market value of the deep learning industry will increase to $60.5 billion by 2025 from $12.3 billion in 2020, an increase in the growth rate of 37.5%. Among industrial sectors, agriculture is one of the slowest to adopt deep learning but has a high potential for its use to combat global food insecurity.

The increasing world population poses a threat to food security. According to the Food and Agricultural Organizations of the United Nations, global food production needs to increase by 70% to provide for 9 billion people by the year 2050 (Askew, 2017). However, this increased food production must be done on about the same amount of farmland used today. The only way to ensure food security is to increase the production of the crops. According to the world food summit in 1996, food security is defined as, “Food security exists when all people, at all times, have physical and economic access to sufficient, safe and nutritious food that meets their dietary needs and food preferences for an active and healthy life” (Godfray et al., 2010). So, ensuring increased food production is not enough for food security. We need to provide nutritious food (Tripathi et al., 2019). We can ensure food security by breeding new cultivars of crops that have higher quality, higher yield, better nutrition, and greater resilience to variable environmental conditions. Food security can also be enhanced by introducing better management systems to maximize the outcome of our food production systems (Jiang and Li, 2020). Scientists have been working relentlessly to introduce new ways of achieving food security, and they believe that the inclusion of technology in agriculture can help us achieve this goal. The study of plant phenotyping can not only help us in designing better crop management systems but also provide new ways of improving crop characteristics, such as yield. Along with increasing food production, we also need to ensure food quality and safety, as well as the economic and environmental sustainability of the food production system. Plant phenotyping plays an important role by informing both crop breeding and crop management.

Plant phenotyping is the assessment of complex plant traits such as growth, development, abiotic and biotic tolerance and resistance, architecture, physiology, ecology, yield, and the basic measurement of individual quantitative parameters that form the basis for complex trait assessment (Li et al., 2014). Recently, there has been significant improvement in plant phenotyping studies. The inclusion of smart farming (Wolfert et al., 2017) and precision agriculture (Gebbers and Adamchuk, 2010) have allowed the extension of conventional tools and provided farmers aware systems that are autonomous, context-aware, and can be controlled remotely. Big data technology is playing an essential role in this development (Wolfert et al., 2017), and deep learning models are an integral part of it. Recently, there has been an increased interest in deep learning-based plant phenotyping studies due to their superiority over traditional analysis (Chandra et al., 2020; Jiang and Li, 2020; Ren et al., 2020; Kolhar and Jagtap, 2021; Arya et al., 2022).

We use deep learning models to process a large amount of data to build decision systems without properly understanding the decision-making process (Guidotti et al., 2018). In May 2018, the General Data Protection Regulation law was enforced in the European Union and European Economic Area, which indicates that whoever uses automated systems for profiling and/or decision making has to ensure fairness, transparency and provide anyone with a meaningful explanation of the logic used (EU, 2018). As a result, in recent years, there has been significant growth in the study of explainable deep learning models (Biran and Cotton, 2017; Preece, 2018; Vilone and Longo, 2021), more commonly known as Explainable AI (XAI). XAI is being adopted in different fields of study to explain existing models and develop better models (Tonekaboni et al., 2019; Bhatt et al., 2020; Bai et al., 2021; Gulum et al., 2021; Thomas et al., 2021). Table 1 shows the number of publications each year where the studies used deep learning or XAI for plant phenotyping. We retrieved the data from a PubMed search with the keywords machine learning or deep learning and plant phenotyping, and machine learning or deep learning and plant phenotyping and explainability. We considered other keywords (e.g., explainable AI, transparent AI, XAI) during our search, however, we found that the combination of the mentioned keywords returned the most relevant papers. From Table 1 it is evident that more plant phenotyping studies are adopting deep learning. Although the trend shows that more researchers are using XAI for studies, it is still in its early stages.

**Table 1:** Evolution of the number of publications that refers to deep learning in plant phenotyping and XAI in plant phenotyping.

	**2016**	**2017**	**2018**	**2019**	**2020**	**2021**	**2022**
Machine learning, deep learning, plant phenotyping	13	39	48	80	113	135	168
Machine learning, deep learning, plant phenotyping, explainability	3	3	3	4	6	9	13

Darker red and blue represent higher numbers.

The availability of deep learning algorithms has allowed plant scientists to easily incorporate them into their studies and achieve impressive results on challenging problems. However, due to the black-box nature of deep learning models, plant scientists are sometimes unaware of how such results were achieved. As a result, any mistake in the development of a deep learning model remains unnoticed, which might affect the generalisability of the models. This black box nature also limits the ability of plant scientists to understand the relation between the results of a deep learning model and plant traits. Additionally, the lack of understanding makes it difficult for scientists to explain the results of a deep learning model to the user of the resulting tool or services. XAI has the capability of explaining the decisions of a model. Such explanations can be utilized to better understand the model and relate the features detected by the model to the plant traits.

The motivation of this study is to provide a detailed overview of the recent developments in XAI techniques so that researchers working in plant phenotyping are able to develop explainable and transparent deep learning models. We thus focus on achieving the following objectives.

- Review XAI techniques in deep learning studies: This objective focuses on reviewing existing XAI techniques that may assist researchers in interpreting the predictions and explaining the decisions of a deep learning model. This comprehensive review is to help researchers understand the capabilities of different XAI techniques and the appropriate contexts and modalities for their application.
- Explore the application of deep learning in plant phenotyping: This objective focuses on reviewing popular studies in the field of plant phenotyping that have utilized deep learning models, to provide researchers insights into how deep learning is advancing and improving the outcomes of plant phenotyping studies.
- Investigate the limitations and opportunities of XAI techniques in plant phenotyping: This objective includes a review of plant phenotyping studies utilizing XAI techniques. Also, an exploration of how XAI can reveal plant traits by analyzing large plant datasets, as well as help build trust in the predicted traits for use in downstream experiments and sections in breeding programs.

## 2. Background

Researchers have been using intelligent and automated systems for a long time. Moore and Swartout (1988) was the first to point out the necessity of explaining intelligent systems. During the same period, Swartout (1983) developed a system to justify the decision of a code, and used it to explain the behavior of a Digitalis Therapy Advisor. However, Van Lent et al. (2004) was the first to use the term XAI to describe the architecture and reasoning capabilities of a U.S. Army's training system. We have come a long way since then. In this section, we first define XAI, then describe its categories, and finally provide an overview of existing XAI techniques.

Despite the recent development, researchers are still divided on the definitions and terminologies used in XAI techniques. XAI refers to tools, techniques, and methods that help humans understand, interpret and trust the decision of an artificial intelligence model (Adadi and Berrada, 2018; Gunning et al., 2021; Vilone and Longo, 2021). XAI methods are also commonly known as interpretable AI, which is a system where the users can interpret how the input and output are mathematically related (Bellucci et al., 2021). Although the tools provide an interpretation of the features a model uses for its decision-making, the terms interpretability and explainability are used interchangeably in the literature. However, Adadi and Berrada (2018) stated that the interpretable model could be considered explainable if humans can understand the operation of the model. Additionally, Linardatos et al. (2020) mentioned that the depth of human understanding of the internal procedure of a model depends on the quality of the explanation. In the context of machine learning, explainability techniques summarize the behavior of the models and describe the system's internal reasoning and dynamics (Gilpin et al., 2018). On the other hand, interpretability is considered to be the degree to which a human can understand the reason for a machine learning model's decision (Miller, 2019). Although the techniques described in the following sections are more concerned with interpretation than explanation, for consistency with other literature, we address them as XAI techniques.

The ethical aspects of a deep learning model have given rise to the term responsible AI. Explainability techniques that deal with the social impact and ethical and moral obligations are called responsible AI (Dignum, 2017; Arrieta et al., 2020). Transparent AI is another common term that is related to explainability techniques. Lipton (2018) considered an AI model transparent if a domain expert is able to calculate the model's prediction in a reasonable time using the input data and model parameters. Although this definition applied to linear models, through transparent AI, researchers try to build non-linear models whose decisions can be explained even when they behave unexpectedly (Lyons, 2013; Larsson and Heintz, 2020).

### 2.1. Categories of XAI techniques

**Figure 1:**
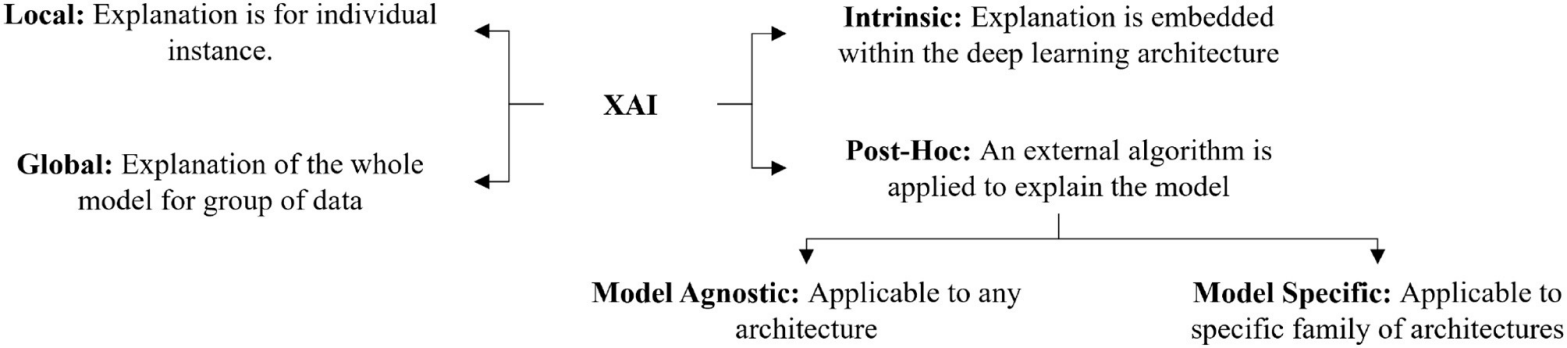
Graphical representation of the categories of XAI techniques.

There are several ways to categorize different XAI techniques. The most common ways of categorizing XAI techniques are based on the scope of explanation, the level of implementation, and the transferability of algorithms. Figure 1 shows an overview of the different categories of XAI Techniques.

#### 2.1.1. Global and local

Depending on the explanation's scale, several researchers have categorized the explanation as either global or local (Ribeiro et al., 2016; Adadi and Berrada, 2018; Ghorbani et al., 2019b; Ibrahim et al., 2019; Burns et al., 2020; Mohseni et al., 2021). Global explainability techniques provide a comprehensive explanation of how the model works (Liu et al., 2016; Nguyen et al., 2016; Kim et al., 2018; Ghorbani et al., 2019b; Ibrahim et al., 2019). Although global explanations allow the user to gain an overview of the model and help them quickly identify what features might be impacting the model's performance, the global explanation's efficiency is largely dependent on the complexity of the model. On the other hand, the explanation of the model's decision for a specific instance is considered to be a local explanation (Ribeiro et al., 2016; Lundberg and Lee, 2017; Mehdiyev and Fettke, 2021; Huang et al., 2022). Local explainability techniques can easily be adapted for a complex model but lack generalizability. We can use local explainability techniques to help users understand how a model performs for different examples. In our study, we found that local explanation techniques are more popular than global explanation methods. This may be due to the complexity of the process of how a deep learning model makes a decision. Designing an algorithm to explain the model's decision for a single example is generally easier than designing the algorithm considering the decisions for all the instances. In future, the innovation of global XAI techniques can provide detailed insight into the inner working of the models.

#### 2.1.2. *Post-hoc* and intrinsic

Another important categorization of XAI techniques relates to how the XAI method is implemented in the model. It can either be implemented within the model or implemented as an external algorithm (Samek and Müller, 2019; Danilevsky et al., 2020; Das and Rad, 2020; Belle and Papantonis, 2021). XAI techniques that are model dependent and embedded within the model, are called intrinsic XAI techniques (Schetinin et al., 2007; Grosenick et al., 2008; Caruana et al., 2015; Letham et al., 2015). Intrinsic techniques are usually applicable to linear models. Such techniques are nontransferable, and any change in the architecture can cause the XAI technique to fail. A more popular way of explaining deep learning models is called *post-hoc* explanations. An XAI technique is considered *post-hoc* if an external explainer is used on a trained model to understand the reasoning behind its decision (Bach et al., 2015; Lundberg and Lee, 2017; Tan et al., 2018; Brunese et al., 2020; Soares et al., 2020). We found that most XAI techniques designed for deep learning models are *post-hoc*. The intrinsic explanations require the techniques to be embedded within the model. In deep learning models, there are a large number of parameters which are distributed over different layers and the layers are non-linearly correlated to each other. As a result, it is difficult to develop intrinsic explanations for deep learning models. So, researchers tend to use *post-hoc* external algorithms to explain models.

#### 2.1.3. Model agnostic and model specific

Depending on the transferability of an XAI method, it can either be model-specific or model-agnostic. A model-agnostic XAI technique does not depend on the model architecture. These methods can be applied to any deep learning model (Ribeiro et al., 2016, 2018; Li et al., 2018). Model-agnostic techniques provide a trade-off between the accuracy of the explanation and generalizability. Explanations produced by a model-specific XAI technique are restricted to a specific model or dataset. Such techniques are not transferable to other models (Hendricks et al., 2016; Lapuschkin et al., 2016; Soares et al., 2020; Agarwal et al., 2021). As a result, they tend to be more accurate in explaining a specific model's decision. So, researchers prefer model-specific techniques rather than model-agnostic ones.

### 2.2. Overview of XAI

The first deep learning algorithm was developed by Ivakhnenko (1968), where the authors proposed a multilayer artificial neural network that was capable of updating its own architecture and complexity. Fukushima (1988) was the developer of the first Convolutional Neural Network (CNN) model, Necognition, that used reinforcement learning for training and used pooling layers and convolutional layers. Inspired by Necognition, LeCun et al. (1989) proposed ConvNet, a deep learning model using supervised training and backpropagation for analyzing image data. Since then, researchers have developed more complex and more capable models, such as Generative Adversarial Networks (Goodfellow et al., 2014), Inception (Szegedy et al., 2015) architectures, and Transformers (Vaswani et al., 2017). In comparison to the development of more accurate and efficient models, there have been far fewer prior works focusing on explaining such complex models. However, there has been a recent increase in interest in model explainability techniques (Arrieta et al., 2020).

#### 2.2.1. Analysis of existing XAI review articles

The notions used in XAI studies lack a proper and consistent definition, and therefore researchers without domain knowledge may find it difficult to understand XAI concepts. To close the gap of understanding, a popular structure for review papers on this topic are to introduce notions, taxonomies, and relatable concepts and then review the related articles (Adadi and Berrada, 2018; Gilpin et al., 2018; Arrieta et al., 2020; Das and Rad, 2020; Vilone and Longo, 2020). Arrieta et al. (2020) reviewed 400 articles and proposed a novel definition of explainability, and emphasized that XAI is necessary to ensure security. In machine learning, fairness is considered a subsection of machine learning interpretability and addresses the social and ethical consequences of machine learning algorithms (Tian et al., 2022). Linardatos et al. (2020) studied the fairness of machine learning models where the authors mentioned that researchers favor groups of individuals with different attributes over ensuring individuals are treated similarly; thus, the importance of individuals is often ignored. Chatzimparmpas et al. (2020) reviewed the studies of visualization and visual interpretation of machine learning models by categorizing them and qualitatively representing them, and finally identified the research gaps in the utilization of XAI and proposed ways of integrating them. Liu et al. (2017) provided an overview and summary of interactive models in deep learning, which can help users better explain models by interacting with them. In their study, Adadi and Berrada (2018) described the need for XAI in different fields and the implication of adapting it to the current AI systems. A methodological approach to evaluate XAI based on a taxonomy of interpretability was proposed by Gilpin et al. (2018). Preece (2018) reviewed the latest XAI techniques and demonstrated that the fundamental problems associated with machine learning algorithms have a long history and the elements of earlier research can help advance today's XAI models.

There are also domain-specific reviews of XAI studies (Danilevsky et al., 2020; Jiménez-Luna et al., 2020; Tjoa and Guan, 2020). Danilevsky et al. (2020) reviewed the recent advancement of XAI techniques in natural language processing and found that researchers prefer local XAI over global ones. This preference is influenced by the increased usage of the black box models in comparison to the white box models. Tjoa and Guan (2020) studied the state-of-the-art XAI techniques and suggested how the techniques can be utilized in the medical domain. The authors emphasized the importance of accountability and transparency in deep learning models within the medical sector and how XAI can help ensure these attributes. In the review conducted by Jiménez-Luna et al. (2020), the authors explore the application of XAI in the field of drug discovery and highlight the potential of XAI techniques to address the challenges faced in this domain. They emphasize that despite the popularity of deep learning models, the mathematics behind the model still remains elusive to most researchers, and XAI can help expand our understanding by providing interpretability and insights into these models. In Table 2, we have summarized the reviews on explainability techniques, which can help us understand the structure of the reviews and the techniques discussed.

**Table 2:** Overview of the review of XAI techniques.

**References**	**Categories discussed**	**Additional information and findings**
Belle and Papantonis (2021)	1. Perspectives on explainability	1. Examples of how data scientists can apply XAI techniques in practice.
	2. Exploring explainable machine learning	2. Suggested that researchers need to focus on building trust in the explanations themselves.
	3. Transparent models	
	4. Opaque models	
	5. Explainability approaches	
Vilone and Longo (2020)	1. Review articles	1. Discussion on the boundaries of XAI
	2. Theories	2. Proposed a framework that ensures human incorporation in the development of XAI
	3. Methods	
	4. Evaluations	
Das and Rad (2020)	1. Scope of explanation	1. Presented historical timeline for XAI studies
	2. The difference in methodology	2. Provided mathematical overviews and algorithms of seminal works in the field of XAI
	3. Implementation level	3. Provided reference to some popular XAI software
	4. Evaluation methodologies	4. Suggested that current XAI utilize model agnostic and *post-hoc* techniques in additive and surrogate models
Linardatos et al. (2020)	1. Explain black-box model	1. Discussed the effect of XAI in bias study
	2. Create white-box model	2. Provided links to the programming implementation of XAI techniques
	3. Enhance fairness	3. Proposed a taxonomy of the existing machine learning interpretability methods
	4. Analyze the sensitivity of model predictions	
Chatzimparmpas et al. (2020)	1. Visual analytics	1. Review of visual analytics in machine learning
	2. General ML models	2. Future opportunities
	3. Predictive visual analytics	3. Research opportunities
	4. Interactive machine learning	4. Review of reviews
	5. Deep learning	
	6. Dimensionality reduction	
Danilevsky et al. (2020)	1. Categorization of explanations	1. Discussion is specific to natural language processing
	2. Aspects of explanations	2. Emphasized the importance of including humans in the development of XAI techniques
	3. Explanation quality	
Jiménez-Luna et al. (2020)	1. Feature attribution methods	1. Discussion is specific to drug discovery
	2. Instance-based approaches	2. Provides an overview of the packages
	3. Graph-convolution-based methods	3. Suggested that XAI can help avoid human bias in the formulation of complex pharmacological hypotheses
	4. Self-explaining approaches	
	5. Uncertainty estimation	
Xu et al. (2019)	1. Making the parts in DNN Transparency	1. Discussed the history of XAI
	2. Learning semantic graphs from existing DNNs	2. Suggested that deep learning models should be transparent for mission-critical tasks
	3. Generation of explanations	
Samek and Müller (2019)	1. Explaining with surrogates	1. Discussed the necessity of XAI
	2. Explaining with local perturbation	2. Showed that current evaluation techniques are inadequate for evaluating the quality of explanations
	3. Propagation-based	
	4. Meta explanations	
	1. Transparent machine learning models	1. A brief discussion of future opportunities
	2. *Post-hoc* explainability techniques for machine learning models	2. Discussion of bias and fairness in XAI
	3. Toward responsible AI	3. Unvelied that XAI has the potential to compromise the data when explaining the models
Adadi and Berrada (2018)	1. XAI methods taxonomy: overview of the existing XAI methods	1. Discussion of potential domains where XAI can benefit existing AI systems
	2. XAI measurement: XAI evaluation techniques	2. Found evidence of lack of formalism and insufficient human role in the development of XAI
	3. XAI perception: role of humans in XAI	
	4. XAI antithesis: works that challenge XAI techniques	
Preece (2018)	1. Explanation in classical AI systems	1. Proposed a framework that researchers can follow to develop XAI techniques
	2. Interpretability in ML-based AI systems	2. Emphasized the necessity of automated tools to easily generate explanations
	3. An explainable AI framework	
Gilpin et al. (2018)	1. Explanations of deep network processing	1. Review of related works in various domains
	2. Explanations of deep network representations	2. Evaluation techniques
	3. Explanation-producing systems	3. Observed that current XAI techniques are siloed and algorithms should be developed to incorporate multiple techniques in a single explanation
Liu et al. (2017)	1. Understanding	1. Review of visualization tools for XAI
	2. Diagnosis	2. Suggested that it is important to quantify the uncertainty of the XAI techniques to gain human trust
	3. Refinement	
Tjoa and Guan (2020)	1. Perceptive interpretability	1. Discussion is specific to the medical domain
	2. Interpretability via mathematical structure	2. Discussion on general XAI techniques and XAI in the medical domain follows the same categories
	3. Other perspectives to interpretability	3. Found that in the medical domain, a unified notion of interpretability is elusive and requires more comparative studies between the performance of XAI techniques

#### 2.2.2. Methods of XAI

To study the prospect of XAI techniques in plant phenotyping, we believe it is important to have knowledge of the existing XAI techniques. We have categorized the existing XAI techniques into six different categories based on the explanation generated by the techniques. Table 3 provides an overview of the models discussed in this section.

**Table 3:** Overview of XAI techniques.

**References**	**Local/**	**Model specific/**	**Intrinsic/**	**Dataset**	**Models examined**
	**global**	**model agnostic**	***post-hoc***		
Che et al. (2015)	G	MA	PH	Khemani et al. (2009)	1. Deep feed-forward neural network
					2. Stack denoising autoencoder
					3. Long Short-Term Memory
Ribeiro et al. (2016)	L	MA	PH	Blitzer et al. (2007)	1. InceptionV3
					2. Word2vec
Ribeiro et al. (2018)	L	MA	PH	Ribeiro et al. (2018)	InceptionV3
Lundberg and Lee (2017)	L, G	MA	PH	Deng (2012)	CNN: 2 Conv layer, 1 FCN layer
Lapuschkin et al. (2016)	L	MS	PH	Everingham et al. (2015)	1. BVLC reference classifier
					2. VGG16
					3. GoogleNet
Hendricks et al. (2016)	L	MS	PH	Wah et al. (2011)	Proposed model combining VGG16 and LSTM
Zhou et al. (2016)	L	MS	PH	Russakovsky et al. (2015)	1. Network in network
					2. GoogleNet
					3. VGG16
Selvaraju et al. (2017)	L	MS	PH	Russakovsky et al. (2015)	1. VGG16
					2. AlexNet
					3. Neuraktalk2
Chattopadhay et al. (2018)	L	MS	PH	1. Russakovsky et al. (2015)	VGG16
				2. Everingham et al. (2015)	
Simonyan et al. (2013)	L	MS	PH	Berg et al. (2010)	ImageNet Classification with deep sonvolutional neural networks
Li et al. (2018)	G	MA	PH	Krizhevsky and Hinton (2009b)	1. ResNet
					2. DenseNet
					3. VGG16
Bach et al. (2015)	L	MA	PH	1. Everingham et al. (2015)	1. Shallow CNN
				2. Deng (2012)	2. Caffe open source pack-age
Ghorbani et al. (2019b)	G	MA	PH	Russakovsky et al. (2015)	InceptionV3
Ibrahim et al. (2019)	G	MA	PH	1. Synthetic data	Shallow CNN
				2. Dua et al. (2017)	
Agarwal et al. (2021)	G	MS	PH	1. Saeed et al. (2011)	Shallow CNN
				2. ProPublica (2016)	
Zeiler and Fergus (2014)	L	MA	PH	1. Fei-Fei et al. (2006)	ImageNet Classification with deep convolutional neural networks
				2. Griffin et al. (2007)	
				3. Everingham and Winn (2011)	
				4. Deng et al. (2009)	
Zintgraf et al. (2017)	L	MA	PH	Deng et al. (2009)	1. AlexNet
					2. GoogleNet
					3. VGG16
	L	MA	PH	1. Krizhevsky and Hinton (2009b)	1. ImageNet Classification with deep convolutional neural networks
				2. Krizhevsky and Hinton (2009a)	2. Network in network
				3. Russakovsky et al. (2015)	
Burns et al. (2020)	L	MA	PH	Deng et al. (2009)	1. InceptionV3
					2. Bidirectional encoder representations from transformers
Soares et al. (2020)	G	MS	PH	Nageshrao et al. (2019)	-
Angelov and Soares (2020)	G	-	I	1. Rezaei and Terauchi (2013)	Proposed the model
				2. Griffin et al. (2007)	
				3. Fei-Fei et al. (2006)	
				4. Yang et al. (2020)	
Lee et al. (2019)	L	MA	PH	Wang et al. (2017)	1. VGG16
					2. ResNet50
					3. InceptionV3
					4. Inception-RecNet-v2
Brunese et al. (2020)	L	MA	PH	Cohen et al. (2020)	VGG16
Assaf and Schumann (2019)	L	MS	PH	Energy consumption of photovoltaic power plant	Proposed the model
Nigri et al. (2020)	G	MS	PH	1. Weiner et al. (2013)	1. AlexNet
				2. Ellis et al. (2009)	2. VGG16
					3. ResNet50
Erion et al. (2019)	G	-	I	Krizhevsky and Hinton (2009b)	VGG16

L, Local; G, Global; MA, Model Agnostic; MS, Model Specific; PH, Post-Hoc; I, Intrinsic; and Dataset refers to where the dataset used in the study was first proposed.

##### 2.2.2.1. Visualization-based XAI

Visualizing the decisions made by different parts of a deep learning model or visualizing the learned features that contributed to the prediction of an instance is a popular way of explaining a model. Layer-Wise Relevance Propagation (LRP) is a deep learning model explanation technique which helps to quickly find relevant features responsible for the prediction (Bach et al., 2015). LRP can be used for various deep learning architectures and data types, which makes it popular in XAI. Lapuschkin et al. (2016) analyzed the reasoning behind the prediction of a Fisher Vector and Deep Neural Network (DNN) models. The authors used a heat mapping technique to find the pixels contributing to the prediction which can be used to determine whether or not the model uses relevant features for prediction. Hendricks et al. (2016) combined natural language processing with visual analytics to generate explainable systems that humans easily understand. The authors trained to separate systems where the first system was trained for image classification, and the second system was trained to generate text descriptions of the discriminating features of a class. The loss function is an essential part of a deep learning model. Instead of optimizing the loss functions, Li et al. (2018) proposed a loss landscape visualization technique that can better capture the sharpness and flatness in the landscape. They also showed that the visualization technique is more intuitive and easily understandable than other techniques. Gradient-weighted class activation mapping (GradCAM) uses the class-specific gradient flowing through a CNN's final convolutional layers to visualize the input's important features in a saliency map. Assaf and Schumann (2019) used GradCAM to explain a model trained on multivariate time-series data.

##### 2.2.2.2. Saliency map based XAI

GradCAM++ is an extension of GradCAM (Chattopadhay et al., 2018). It allows for the visualization of multiple objects of the same class in the image as it uses a weighted combination of positive partial derivatives of the last convolutional layer to generate the visualizations. Ghorbani et al. (2019b) proposed a concept-based approach called Automated Concept-based Explanations (ACE) which can help reveal whether a model's prediction correlates to any unwanted features. Zintgraf et al. (2017) proposed a saliency map generation technique, where for each prediction, a relevance value is assigned to each input feature with respect to the class. Simonyan et al. (2013) created saliency maps representing the discriminative features of a class by passing a single backpropagation through a CNN. Guided Backpropagation (GBP) is a gradient-based visualization technique that allows the visualization of the image features that activate the neurons in a deep learning model (Springenberg et al., 2014). In the field of medical science, Lee et al. (2019) proposed a deep learning-based explainable acute intracranial hemorrhage system that generated saliency maps showing the relevant features in a class.

##### 2.2.2.3. Surrogate models

A surrogate model is a simple model that is used to explain a complex model. Local Interpretable Model-agnostic Explanation (LIME) (Ribeiro et al., 2016) is a popular example of a surrogate model, that can help identify regions in the input essential for the prediction. Ribeiro et al. (2016, 2018) proposed extensions of LIME called Sub-modular Pick LIME (SP-LIME) and Anchors, respectively. Shapley additive explanations (Lundberg and Lee, 2017) is another example of using the surrogate model to explain a deep learning model, which assigns an importance value to each feature for an instance. Tan et al. (2018) proposed Distill-and-Compare that can explain an inaccessible black-box model by training a model with labeled data with risk factors and then training another model to predict the outcome. Che et al. (2015) used knowledge distillation from deep learning models to explain the features and prediction rules with gradient boosting trees. Soares et al. (2020) proposed a rule-based surrogate XAI model for deep reinforcement learning where the results of the reinforcement learning model are replicated with an if-then rule-based model.

##### 2.2.2.4. Attribution mapping

The benefit of a global explanation is that it allows for a description of the neural network using a single set of features. Global Attributions Mapping proposed by Ibrahim et al. (2019) allows granularity of analysis by increasing or decreasing the size of the subpopulation. Erion et al. (2019) developed a framework called attribution prior using the feature attribution method that enforces a deep learning model to train based on prior expectations and allows encoding of human intuitions without the necessity of knowing unimportant features beforehand.

##### 2.2.2.5. Additive models

Generalized additive models are a class of linear model (Lou et al., 2012) that combines multiple models where each model is trained with individual features. A drawback of this model is that it fails to work with non-linear functions. An extension of this approach was proposed by Agarwal et al. (2021), called Neural Additive Models (NAM), where, a linear combination of neural networks models is used to generate a prediction. NAM can help generate an explanation of individual features for a prediction.

##### 2.2.2.6. Perturbation-based models

In a perturbation-based XAI technique, explanations are generated by probing a trained model with different variations of the input data. The Interpretability Randomization Test and the One-Shot Feature Test proposed by Burns et al. (2020) are perturbation-based XAI methods. The intermediate layers of a CNN model were visualized by Zeiler and Fergus (2014). The authors hide different parts of an input image and used a Deconvolutional Neural Network to regenerate the input. The saliency maps generated by this process represent the features responsible for the activation of the feature map. Angelov and Soares (2020) proposed a generative explainable deep learning model that is automatically built from the training data without defining parameters, problem-specific thresholds, and intervention. The swap test is an explainable deep learning model that generates heatmaps representing the area of interest in the MRI images of Alzheimer's patients (Nigri et al., 2020).

Recently several XAI techniques have been developed and applied in different fields of studies (Cabitza et al., 2017; Bhatt et al., 2020; Bai et al., 2021; Gulum et al., 2021; Puyol-Antón et al., 2021; Thomas et al., 2021). In our review, we found that researchers prefer the visualization-based XAI and saliency map-based XAI techniques over others. These models are capable of explaining the decisions of a model through the generation of different visualization maps and images, therefore researchers find them easy to understand and adopt. In Table 3 we summarize the XAI techniques described above. In addition, Table 3 offers valuable insights into the dataset employed and the deep learning models utilized in each study, along with the scope and type of the explanations. Specifically, we provided information regarding where the dataset was first proposed, enabling researchers to acquire comprehensive knowledge about its intricacies.

An XAI technique aims to generate explanations that can help humans understand how the decisions are made in a deep learning model (Gerlings et al., 2020). Existing XAI techniques are more focused on explaining the models, or variables responsible for the decisions of a model, and there is a lack of XAI techniques that utilize the explanations to improve the model's performance. Furthermore, although XAI techniques are designed to explain the decision of deep learning models, they are used less frequently in deep learning studies than expected. The inadequate adoption of the XAI techniques can be attributed to the lack of proper XAI evaluation techniques, the unreliability of XAI techniques, and the unavailability of XAI platforms and tools. Although researchers have proposed a few XAI evaluation techniques (Arras et al., 2016, 2017; Samek et al., 2016; Ancona et al., 2017; Adebayo et al., 2018a,b; Alvarez Melis and Jaakkola, 2018; Mohseni et al., 2018; Ribeiro et al., 2018; Yang and Kim, 2019; Holzinger et al., 2020), the techniques usually suffer from limited generalizability and inconsistency. Furthermore, researchers found that the explanations for similar models vary from one XAI technique to another thus promoting reliability concerns (Adebayo et al., 2018b; Ghorbani et al., 2019a; Kindermans et al., 2019; Weerts et al., 2019). Finally, compared to the deep learning models, there is a lack of platforms and resources that can help researchers easily adopt XAI in their studies. Explaining the decisions of a deep learning model is crucial for fostering user trust and facilitating transparency. This practice can enable researchers from diverse domains to confidently integrate deep learning models into their studies, ensuring a robust foundation for their investigations. We believe the implementation of XAI techniques in the analysis of plant phenotyping data can help plant scientists develop a better understanding of data-derived plant traits. We explore this topic in the following section.

## 3. Explainable AI and plant phenotyping

Plant phenotyping is the study of characterizing and quantifying the physical and physiological traits of a plant (Chandra et al., 2020). Plant phenotyping can help us understand plant characteristics like chlorophyll content, leaf surface temperature, leaf size, leaf count, shoot biomass, photosynthesis efficiency, plant growth rate, germination time, and emergence time of leaves (Kolhar and Jagtap, 2021). The results of plant phenotyping studies allow us to develop a better crop management system (Bauer et al., 2019). We can detect plant disease, type of plant, the water content in the plant, and flowering of plants, and take necessary steps if a problem arises (DeChant et al., 2017; Ghosal et al., 2019; Arya et al., 2022). Recently, scientists have started applying deep learning techniques to plant phenotyping studies (Almahairi et al., 2018; Ghosal et al., 2019; Mortensen et al., 2019). Deep learning models can analyze large amounts of data, find previously thought impossible features, and do all these more accurately than ever before. As a result, researchers are increasingly adopting deep learning into their studies. However, deploying deep learning techniques requires domain knowledge of machine learning algorithms as numerous models perform various tasks. It creates a dilemma among plant scientists in deciding which model to choose, how to use it, and how to incorporate the results into their studies.

### 3.1. Deep learning models in plant phenotyping

There are few reviews that look into the advancements of deep learning techniques in plant phenotyping. Jiang and Li (2020), Arya et al. (2022), Chandra et al. (2020), Ren et al. (2020), Kolhar and Jagtap (2021) provide detailed overviews of how deep learning is advancing plant phenotyping studies. Although they used different categorization techniques to discuss the papers, the motivation was to introduce plant scientists to deep learning (Ubbens and Stavness, 2017). A more domain-specific study was conducted by Singh et al. (2018), where the authors reviewed deep learning for plant stress phenotyping and suggested that deep learning models utilizing image data in plant phenotyping hold significant potential for early diagnosis of plant stress. The study conducted by Atkinson et al. (2019) emphasizes the importance of integrating deep learning-based 2D systems with large-scale quantitative genetic data analysis as a pivotal progression in the field of root phenotyping, offering valuable insights for understanding root biology and its implications. The modern deep learning-based plant disease detection techniques were discussed in Lee et al. (2020). The authors found that models trained on disease and independent of crops performed better than crop disease pair, and for transfer learning, a popular concept in plant phenotyping, pre-training with a plant-specific task can help reduce the effect of overfitting. The review on the advancement of deep learning for pest and leaf disease detection by Ngugi et al. (2021) reported a notable challenge faced by the deep learning models used in plant phenotyping is the model's inability to generalize across diverse datasets and field conditions. Hasan et al. (2021) reviewed the machine learning techniques for weed detection and classification and revealed the necessity of a large labeled dataset specifically designed for weed detection to overcome the limitations of the current studies that require pre-trained models to improve the detection accuracy and only utilize the existing small datasets. Danilevicz et al. (2022) addresses the challenges of applying machine learning models for predicting phenotypic traits using genetic markers and presents the advantages and disadvantages of using explainable model structures in plant phenotyping. Additionally, the authors reiterated the necessity of labeled data in plant phenotyping studies and suggested that the model accuracy can be improved if the weights of the existing models are updated by training on new datasets.

To help the researchers understand the capabilities of deep learning models and their usage in plant phenotyping, we reviewed the popular deep learning studies in this area in the following sections. We categorized the papers based on the application of deep learning models.

#### 3.1.1. Classification

Plant disease can severely damage the quality and production of crops. Timely and accurate disease detection can help take proper steps to prevent or stop its spread. A simple LeNet (LeCun et al., 1989) based architecture was used by Amara et al. (2017) to classify banana disease using the PlantVillage dataset. Despite its simple architecture, the model performed well under varying conditions.

**Figure 2:**
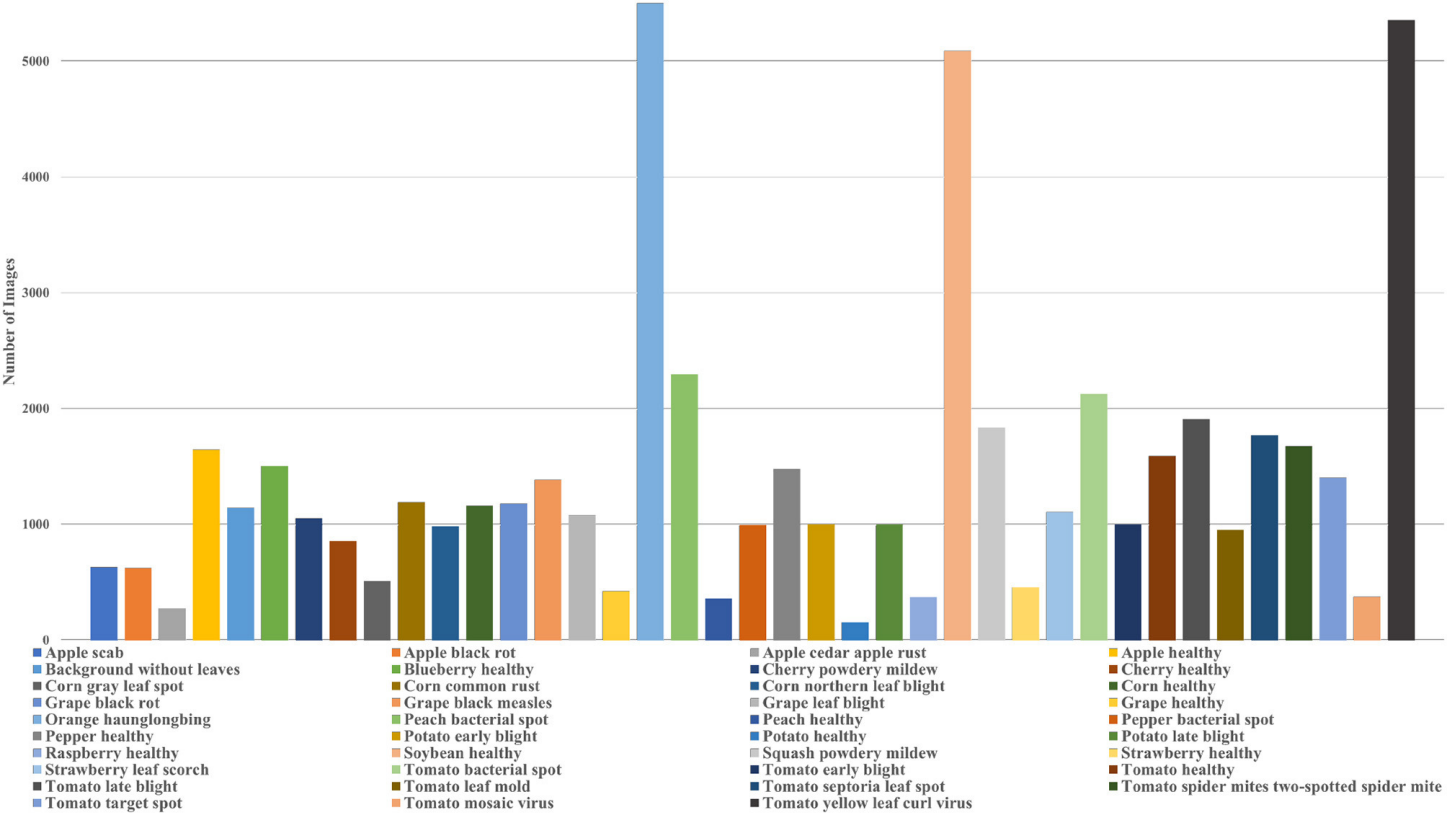
Plot of the distribution of images in different classes of the PlantVillage dataset.

In plant phenotyping, the most popular dataset for classification tasks is the PlantVillage dataset. The dataset contains images of leaves of 14 crop species and 26 diseases. The dataset consists of colored, grayscale, and segmented images of the leaves which were captured in a controlled environment. There are 39 classes in the PlantVillage dataset and the distribution of images among different classes is shown in Figure 2 which also provides information about the different diseases and species covered in the dataset. In their paper, Mohanty et al. (2016) introduced the dataset and also classified the images using AlexNet and GoogleNet. The authors demonstrated the models' performance for different training and test combinations and showed that the models performed exceptionally well. However, the classification of their best-trained model dropped to 31% when tested on field data. Such results might be because images of individual leaves in the dataset were taken in controlled conditions and against a constant background. So, the trained models failed to generalize and performed inadequately when there were changes in illumination, background, or number of leaves. However, this dataset is still used frequently in plant phenotyping and helps scientists develop their models.

DeChant et al. (2017) proposed a deep learning-based framework capable of detecting northern leaf blight-infected maize plants from images acquired by unmanned aerial vehicles (UAV). The framework consists of training several models and combining their results for prediction. At first, the authors trained five deep learning models on image patches to detect the presence of lesions and achieved 94% accuracy. Next, they generated heatmaps showing the probability of infection of every region in an image using the trained models. Finally, they trained another CNN model to classify whether there were infected leaves in the image and the inclusion of the heatmaps increased the classification to 97.8%.

A different approach for analyzing the plant phenotyping traits was adopted by Taghavi Namin et al. (2018). They proposed a CNN and Long Short Term Memory (LSTM) based classifier where the plant growth information was used for the classification. A sequence of plant images representing different stages of growth was used as the input for the CNN model. The CNN model extracted features of an individual plant and passed them on to LSTM. LSTM analyzes this sequence of features by considering the temporal features and using them to classify plants. CNN-LSTM can use the plant's growth information and model the phenotypic and genotypic information. The combination of CNN and LSTM helped the authors achieve 93% accuracy in comparison to 76.8% accuracy by using just CNN. A similar structure was adopted by Ubbens et al. (2020) to detect and quantify a plant's response to the treatment.

Lodging is a state of the crop where it bends and does not return to its original state, often the shoot lays on the ground and is subject to disease and decomposition. LodgeNet is a CNN classifier that can classify images of lodged crops from five spectral channel orthomosaic images of canola and wheat plants (Mardanisamani et al., 2019) where the images are captured using UAVs. In LodgeNet, a seven-layer CNN model is used to extract texture features from the orthomosaic images. In addition, two texture feature extraction algorithms (local binary patterns and gray-level co-occurrence matrix) are also used to extract additional features. Features extracted from the CNN and the texture descriptors were combined to train a deep learning classifier and achieved 97.70 and 99.06% accuracy for wheat and canola, respectively. The results of the LodgeNet were compared with other popular deep learning models and the authors reported an improvement of classification accuracy by 8.84%. The authors concluded that simple deep learning models are capable of performing as efficiently as complex models. The capability of simple deep learning models were also tested by Hati and Singh (2021) where the authors implemented Residual Network (ResNET) based classifiers for the classification of different species and plant health conditions. The results of the ResNet-based classifier were compared with AlexNet and ResNet provided 16% higher F1-score than AlexNet.

#### 3.1.2. Regression

In deep learning, regression is used to investigate the relationship between independent variables or features and a dependent variable or outcome (Kuleshov et al., 2018), and segmentation is used to partition an image into different parts or regions depending on the image pixel's characteristics (Haralick and Shapiro, 1985). In a deep learning-based regression, segmentation is usually used to detect the objects and count them. To accomplish this, an annotated dataset is required. The lack of annotated datasets for plant phenotyping magnifies the challenges and limitations faced in this field of study. To resolve this issue, Dobrescu et al. (2017) proposed a ResNet-based leaf counter that only requires the total leaf count per plant. Another interesting contribution is that the authors combined a dataset of different sources and species to perform better than previous models. In the analysis, the authors observed that using a pre-trained ResNet on the ImageNet dataset performed better than training the model from scratch. It shows that transfer learning can be a viable option to resolve the insufficient data problem.

The authors of Aich and Stavness (2017) used a combination of deconvolutional networks and convolutional networks to count rosette leaves. The networks were trained separately but not independently. At first, a segmentation network was trained to generate binary masks representing the leaves in the image. Next, the binary mask and the images were used to train a counting network. The segmentation was developed using SegNet (Badrinarayanan et al., 2017), and the counting network was developed using VGG16. The study's results showed that the proposed network's generalization capability was better than other state-of-the-art leaf counters. Ubbens et al. (2018) also developed a rosette leaf counter using deep learning. The authors also showed that the synthetic 3D plants could be used to generate augmented training data for the deep learning model when the dataset is not large enough.

A deep learning model is initially trained in a weakly supervised training process with few labeled data. Then the trained model is again retrained with unlabeled data. Ghosal et al. (2019) used weak training to develop sorghum head detection and a counting network. The deep learning model was based on RetinaNet (Lin et al., 2017) and ResNet50. In this work, the first stage was to detect the sorghum heads in the image, and the next stage was to count those heads. In addition, a regression model was used to generate bounding boxes around the sorghum heads. To understand the learning of the residual network, feature maps were visualized. The authors considered the visualization a “trust mechanism”, which showed that the model extracted a significant amount of features from the plant head. The authors proposed a counting framework that can work when there is a shortage of labeled data for the deep learning model.

Pound et al. (2017) proposed an hourglass (Newell et al., 2016) based deep learning architecture capable of localizing wheat spikes and spikelets with 4.09 and 0.34% error, respectively. Another important contribution of the paper is the introduction of the ACID dataset which consists of wheat crop images with annotation and labeling. The model could also classify awned wheat with 99% accuracy in the segmented images. The accuracy curve of the model indicates that it achieved peak accuracy at ~200 epochs out of the total 500 epochs of training, raising the possibility of overfitting as the model at the 500th epoch was utilized for the analysis.

#### 3.1.3. Segmentation

Segmentation plays a crucial role in plant phenotyping. In field conditions, the crop or leaf of a plant is usually accompanied by other plant parts. Proper segmentation is often required to detect the object of interest and use the object of interest for other purposes. However, another interesting application of segmentation is to generate annotated datasets. An oil radish growth dataset was presented by Mortensen et al. (2019), which contained images of oil radish collected over weeks. In the study, the authors used the fully connected neural network proposed by Long et al. (2015) for semantic segmentation of the oil radish and other plants and achieved 71.2% mean intersection over union (mIoU). For annotation in the GrassClover image dataset, Skovsen et al. (2019) used a similar network for semantic segmentation of grass and clover from the field images, where both crops were mixed and reached a mIoU of 55.0%. Bernotas et al. (2019) used recurrent neural network (RNN) (Ren and Zemel, 2017) and Mask R-convolutional neural network (Mask R-CNN) (He et al., 2017) for instance segmentation of rosettes and individual leaves to monitor the growth of the plant. Using RNN and Mask R-CNN Keller et al. (2018) also segmented soybean leaf using color-based thresholding, random forest classifier and deep convolutional network and achieved 87.52, 51.24, and 78.65% mIoU, respectively.

Two stages of CNN models were used for citrus plant detection in Ampatzidis and Partel (2019) by utilizing multispectral images from a UAV. In the first stage, a YOLOv3 model was used to detect tree locations in the images. After using computer vision algorithms on the detected trees, another YOLOv3 was used to find trees in the locations that the first model might have missed. These images were then used in a threshold-based algorithm for image segmentation. The proposed method detected trees with 99.8% accuracy, tree gaps with 94.2% accuracy, and estimated individual tree canopy area with 85.5% accuracy. In Vit et al. (2019), object detection and point of interest identification models were introduced to measure the height of a banana tree and the length, width, and aspect ratio of a banana leaf. The models were developed using Mask R-CNN and Faster RNN and obtained an average deviation of 3% for detecting the height of the tree and 7–8% deviation for leaf width and length estimation.

#### 3.1.4. Synthetic data generation

To train deep learning models, a large dataset is required. In plant phenotyping, there is a lack of such datasets. Also, collecting data for the datasets is very time-consuming and costly. So, Generative Adversarial Network (GAN) to synthesize images to mitigate the scarcity of plant images for deep learning is a popular choice. Cycle-GAN, an image-to-image translator developed by Almahairi et al. (2018), has inspired researchers to adopt this in plant phenotyping studies to generate augmented plant phenotyping data.

**Figure 3:**
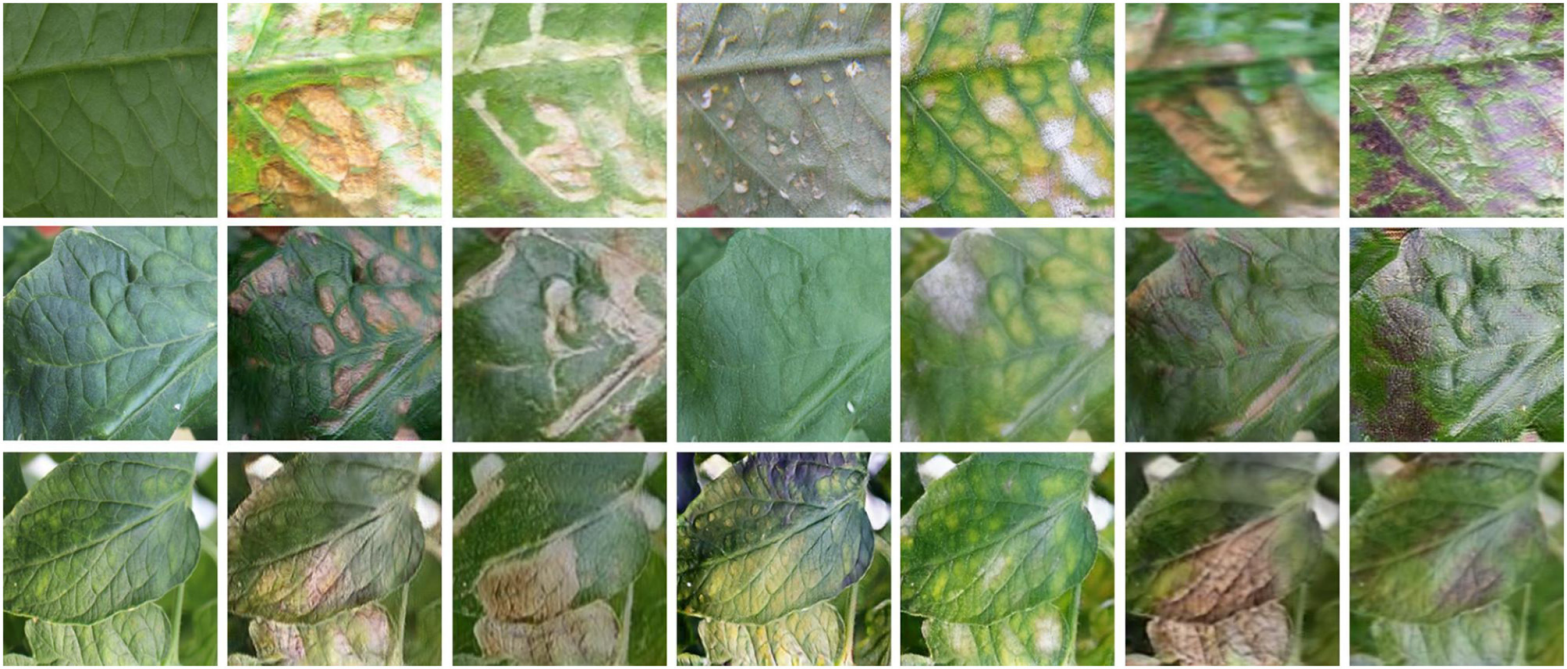
Examples of synthetic images generated by AR-GAN. The leftmost column shows the real images and the rest of the columns show synthetic images with effects of canker, miner, whitefly, powdery mildew, plague, and low temperature (left to right), respectively (Nazki et al., 2020).

Nazki et al. (2020) proposed an unsupervised image-to-image translator called AR-GAN by adopting and improving the concept of CycleGAN. The authors transferred diseased patches from an unhealthy tomato leaf to a healthy tomato leaf. The leaf texture in the synthetic data was consistent with the actual data. The synthetic data was mixed with the training data, and a ResNet model was trained to classify different types of disease. The inclusion of the synthetic data increased the classification accuracy by 5.2%. AR-GAN can help plant phenotyping-based deep learning studies generate cost-effective, larger, and more diverse datasets. Figure 3 shows examples of images generated by AR-GAN.

Cap et al. (2022) showed that AR-GAN failed to synthesize images when the images had a complex background similar to field conditions. So the authors proposed LeafGAN, an image-to-image translating GAN model. Compared to the AR-GAN, LeafGAN utilizes a proposed image segmentation technique, LFLSeg, which segments the leaf from the background. LFLSeg is a CNN that is trained to classify between whole leaf, partial leaf (parts of the whole leaf), and non-leaf. After training the CNN model, for a prediction, Grad-CAM is used to generate a heatmap of the features, which are supposed to be the leaf pixels in this case. The heatmap is turned into a binary mask, and the input image is segmented. The purpose of LFLSeg is to guide the CycleGAN model to focus on the leaf instead of the background. In comparison with CycleGAN, LeafGAN performed superiorly. However, for a new dataset training, the LFLSeg might be difficult. As the partial leaf class was hand-picked, it may require considerable time to generate. The Grad-CAM itself still lacks reliability. Analyzing individual masks generated by Grad-CAM might be impossible for a large dataset.

**Figure 4:**
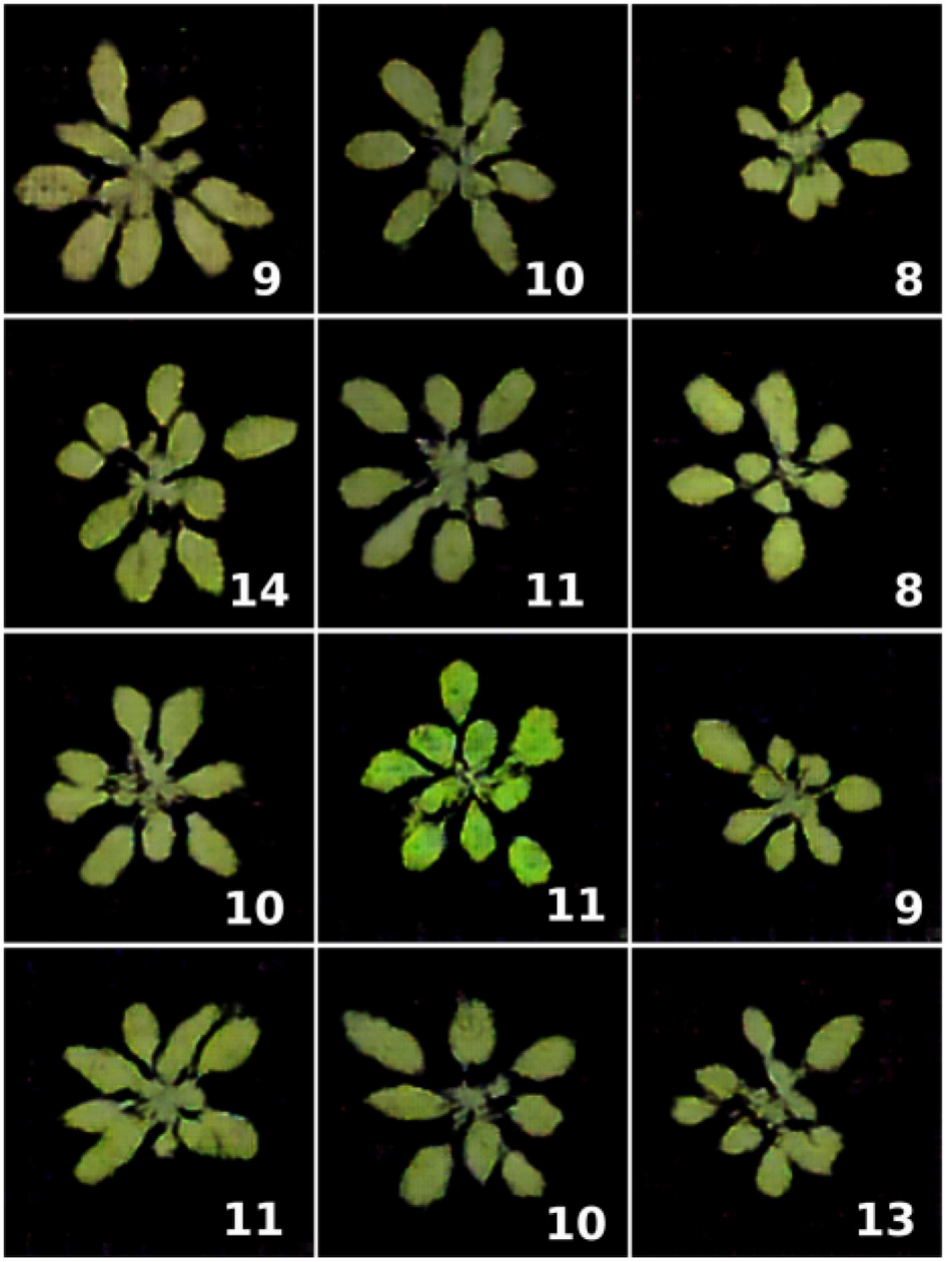
Samples of augmented images generated by ARIGAN. The bottom right represents the number of leaves (Valerio Giuffrida et al., 2017).

To generate rosette plant leaves, Valerio Giuffrida et al. (2017) developed ARIGAN, a GAN-based synthetic image generator. This DCGAN (Radford et al., 2015) based model takes the number of leaves that should be present in the image, uses random noise, and transforms it into the image of a rosette plant with a given number of leaves. The authors used the model to generate 57 images of rosette plants with a varying number of leaves. However, if we look closely at the images in Figure 4, the leaves' variability seems minimal.

#### 3.1.5. Root phenotyping

The focus of this review has been on shoot phenotyping as the majority of the deep learning-based plant phenotyping analyses in the literature have focused on shoot phenotyping. Recently, there has been an extensive increase in deep learning-based root phenotyping. Root phenotyping or the study of plant root architecture and morphology aims at understanding the role of genetic differences in root system architecture in more efficient acquisition of mineral nutrients and water, and response to climate and soil change (Gong et al., 2021). Roots are harder to study *in situ* as roots grow in opaque and complex soil. Along with growing the roots in a transparent media or soil-filled rhozoboxes (Lube et al., 2022), researchers also have focused on developing nondestructive tools and systems to analyze the roots and deep learning has proved to be a great asset for such studies (Mairhofer et al., 2013; Shen et al., 2020).

Segmentation of the plant roots from images captured using different nondestructive imaging techniques is an important part of root phenotyping studies. Thesma and Mohammadpour Velni (2022) proposed a binary semantic segmentation model to segment plant root images of the *Arabidopsis thaliana* using SegNet (Badrinarayanan et al., 2017). Although SegNet was designed to consume less memory during inference time, it achieved comparable segmentation performance with a mean intersection over union of 60.10%. RootNav 2.0 is an automatic root system extraction tool proposed by Yasrab et al. (2019). Along with providing root architecture from images, this autoencoder based deep learning model can locate seeds, and first order and second order root tips with 66.1% mean intersection over union. Seidenthal et al. (2022) proposed an iterative deep learning architecture ITErRoot that allows the refinement of the detected roots during each iteration of model training. The iterative approach of the model can accurately detect the thin and branched root system and generate high quality segmented root images. The authors also proposed a 2D root image dataset with ground truths.

Falk et al. (2020) proposed a high-throughput, cost-effective end-to-end root phenotyping pipeline. The authors developed a low-cost growth chamber to observe the growth of plant roots in a non-destructive manner, a CNN to segment the root structure, and finally used an automatic root imaging analysis tool on the segmented images to study the plant traits. Yasrab et al. (2021) took a different approach to study root phenotyping by developing a GAN model that can forecast the growth of a plant and generate segmentation masks of root and shoot systems by using the forecast data. A simple deep learning classifier was developed by Xu et al. (2022) to classify root types into branch type, taproot type, and an intermediate taproot-branch type. Xu et al. (2022) compared the results of the deep learning model with supervised and semi-supervised traditional machine learning models and concluded that deep learning models perform better.

#### 3.1.6. Deep learning platform for plant phenotyping

Deep learning models demonstrate superior performance compared to shallow and traditional machine learning algorithms across a wide range of tasks due to their ability to learn intricate representations and capture complex patterns in data (Hu et al., 2021; Janiesch et al., 2021; Sarker, 2021). Despite its efficiency, deep learning models are rarely adopted to perform plant phenotyping tasks. The main reasons for the lack of adoption of deep learning in plant phenotyping can be attributed to the absence of proper tools and the lack of large adequately labeled, task-specific plant datasets. Researchers require domain knowledge of the models to build and apply the models to their studies. Furthermore, the development of a generalizable deep learning model relies heavily on the quality and quantity of available data. Substantial computational resources are also necessary to train and deploy deep learning models. To resolve the lack of proper tools, Ubbens and Stavness (2017) developed an open-source deep learning tool called Deep Plant Phenomics. This tool contains pre-trained deep learning models for leaf counting, mutant classification, and age regression. The models were trained for canola and rosette leaves. Hypocotyl UNet by Dobos et al. (2019) is another publicly available tool that can estimate the hypocotyl length in seedlings and can be adapted for different datasets. Nakhle and Harfouche (2021) developed an interactive tutorial with open-source libraries to analyze plant phenotyping data using deep learning models. The authors believe that the tool can benefit early career researchers and students in extracting biologically meaningful information from deep learning models. They also reviewed the tools, techniques, and services available to study plant phenotyping with XAI based image analysis. Plant scientists may consider incorporating these tools into their studies to obtain previously thought impossible results.

In this review paper, we have summarized the plant phenotyping studies utilizing deep learning, as presented in Table 4. The table encapsulates essential details about the purpose of the deep learning model and the specific phenotyping task performed by the researchers. Furthermore, detailed information about the dataset and deep learning model employed in each study has been incorporated into the table. This includes information as to whether the researchers proposed a novel model or dataset, utilized an existing model and dataset, or employed well-established, commonly known datasets and models.

**Table 4:** Overview of the deep learning models in plant phenotyping.

**References**	**Deep learning approach**	**Phenotyping task**	**Dataset**	**Model**
Amara et al. (2017)	Classification	Banana leaf disease detection	PlantVillage	LeNet
Mohanty et al. (2016)	Classification	Disease detection	PlantVillage	1. AlexNet
				2. GoogleNet
DeChant et al. (2017)	Classification	Northern leaf blight detection in maize	Proposed dataset	Proposed model
Taghavi Namin et al. (2018)	Classification	Classification of various accessions of Arabidopsis thaliana	1. Created the dataset	Proposed CNN-LSTM framework
			2. Ara-2013	
Ubbens et al. (2020)	Classification	Detection and quantification of response-to-treatment from images for C4 grass Setaria, sorghum, canola	1. Veley et al. (2017)	Proposed CNN-LSTM framework
			2. Feldman et al. (2018)	
			3. Proposed dataset	
Mardanisamani et al. (2019)	Classification	Lodging classification of wheat and canola	Proposed dataset	Proposed model
Hati and Singh (2021)	Classification	Plant species recognition and health condition identification	Chouhan et al. (2019)	ResNet
Xu et al. (2022)	Classification	Classified root types into branch types	Proposed dataset	Proposed model
Dobrescu et al. (2017)	Regression	Leaf counting in rosette plants	Leaf counting challenge Tsaftaris and Scharr (2017)	ResNet50
Aich and Stavness (2017)	Regression and segmentation	Leaf counting in rosette plants	Leaf counting challenge Tsaftaris and Scharr (2017)	SegNet
Ghosal et al. (2019)	Regression	Sorghum head detection and counting	Guo et al. (2018)	1. RetinaNet
				2. ResNet50
Pound et al. (2017)	Regression	Localizing wheat spikes and spikelets	Proposed ACID dataset	Newell et al. (2016)
Ubbens et al. (2018)	Regression and data augmentation	Augmented *Arabidopsis thaliana* rosette dataset dataset to enhance counting capabilities	1. Ara-2012	1. Ubbens and Stavness (2017)
			2. Ara-2013	2. Mundermann et al. (2005)
Mortensen et al. (2019)	Segmentation	Proposed oil radish dataset and segmented crops	Proposed dataset	Long et al. (2015)
Skovsen et al. (2019)	Segmentation	Segmentation of grass and clover from field images	GlassClover	Long et al. (2015)
Bernotas et al. (2019)	Segmentation	Instance segmentation of *Arabidopsis thaliana*	Proposed dataset	1. RNN
				2. Mask R-CNN
Keller et al. (2018)	Segmentation	Segmentation of soybean leaf	Proposed dataset	DeepLab
Ampatzidis and Partel (2019)	Segmentation	Citrus plant detection	Proposed dataset	YOLOv3
Vit et al. (2019)	Segmentation	Measured the height of a banana tree, and measured the length, width, and aspect ratio of banana leaves in potted plants	Proposed dataset	1. Mask R-CNN
				2. Faster RNN
Thesma and Mohammadpour Velni (2022)	Segmentation and data augmentation	Proposed binary semantic segmentation of plant root	Gaggion et al. (2021)	1. Badrinarayanan et al. (2017)
				2. Wang T.-C. et al. (2018)
Yasrab et al. (2019)	Segmentation	Developed root system extraction tool	1. Pound et al. (2017)	Proposed model
			2. Proposed dataset	
Seidenthal et al. (2022)	Segmentation	Detect and segment root system	Proposed dataset	Proposed model
Falk et al. (2020)	Segmentation	Proposed end-to-end root phenotyping pipeline including root segmentation	1. Oliveira et al. (2010)	Proposed model
			2. Song et al. (2013)	
Nazki et al. (2020)	Data augmentation	Image translator to translate disease images from healthy ones in tomato	1. Proposed dataset	Proposed model
			2. Cityscapes	
			3. Zhu et al. (2017)	
Cap et al. (2022)	Data augmentation	Generates diseased leaf images from healthy ones	Proposed dataset	Proposed model
Valerio Giuffrida et al. (2017)	Data augmentation	Generated images of Arabidopsis Rosette	Leaf counting challenge Tsaftaris and Scharr (2017)	Proposed model
Yasrab et al. (2021)	Data augmentation and segmentation	Forecasted the development of plant roots and generated segmented root images	1. Uchiyama et al. (2017)	1. Proposed model
			2. Wilson et al. (2015)	2. Yasrab et al. (2019)

### 3.2. Explainability in plant phenotyping

As discussed earlier, explainability is becoming an important part of deep learning models as it allows better understanding and provides model optimization capabilities. XAI is needed in plant phenotyping studies as plant scientists need to verify the predictions and be confident in the result so that the model can be applied in practice. Explaining the prediction of the model works as an extra layer of security for plant scientists. In comparison with other fields of study, XAI is still in its earlier stage in plant phenotyping. To the best of our knowledge, the article by Harfouche et al. (2022) is the only other review that discusses XAI in plant phenotyping. However, the focus of the review was to analyze the contribution of XAI in data bias, the infrastructure needed to accommodate XAI in plant phenotyping and the responsibility of humans to utilize XAI. The focus of this study is to discuss XAI techniques and their use cases. In this section, we discuss the studies where XAI was used in plant phenotyping for validation and analysis of results.

#### 3.2.1. Classification of plant phenotyping traits

Classification is an important and common task in plant phenotyping studies concerned with identifying plant species, rating plant traits, or rating disease severity. Consequently, the majority of the XAI techniques in plant phenotyping are tailored for the classification models, enabling researchers to better understand the important features for model development and validate the results produced by deep learning models.

##### 3.2.1.1. Disease classification

Deep learning models have significantly improved the efficiency of detecting plant and leaf diseases from plant images, surpassing the traditional image analysis methods. A stress identification and classification framework for soybean were developed by Ghosal et al. (2018). The proposed framework had two parts. In the first part, a modified version of the CNN proposed by Krizhevsky et al. (2017) was used for stress identification and classification. In the second part, the authors proposed a visualization technique to identify the features in the input image responsible for a prediction. For the visualization, the authors used all the feature maps for all of the healthy leaf images of a low-level layer and calculated a stress activation threshold by computing the probability distribution of the mean activation levels of the feature maps. Next, a feature importance score was assigned to every feature map based on each feature map's mean activation level, computed over those pixels with activation levels above the threshold computed earlier. Finally, based on the importance score, *k*-feature maps were selected, and an explanation map was generated by computing the weighted average of the top-*k* feature maps. The average intensity of the explanation map worked as the percentage of the stress level. The framework worked well for plant stress identification and quantification. The authors mentioned it to be a model-agnostic technique with transfer learning ability. So, it may be used to identify stress in other plants. However, the dataset used in the study was collected in a lab environment where the picture of individual leaves was taken by placing them in front of a black background. In calculating the stress activation threshold, only the foreground pixels were used. In a field condition, several leaves may stay together, and it is hard to distinguish the foreground from the background in such cases. Moreover, we might need a very large dataset to apply the framework to other plants. The plant phenotyping community still lacks such large labeled datasets.

In deep learning-based plant phenotypic studies, researchers often use popular pre-trained models (e.g., InceptionV3, GoogleNet, AlexNet, ResNet) to perform a task (Ngugi et al., 2021). However, these models are designed for large datasets and are very complex in design. Toda and Okura (2019) studied plant disease classification and using visualization techniques showed that complex models do not necessarily contribute to the inference. At first, the authors developed an InceptionV3 (Szegedy et al., 2016) based classifier to classify plant diseases using the Plant Village (Hughes and Salathé, 2015) dataset. Next, the authors used four different classes of visualization techniques, i.e., hidden layer output visualization, feature visualization, semantic dictionary, and perturbation-based visualization, to explain the model. They visualized every layer of the model to understand the learning of the model. Based on the visualization, the authors removed 75% of the network parameters and achieved similar performance. The authors also found that GradCAM (Assaf and Schumann, 2019) and Explanation Map (Krizhevsky et al., 2017) were the most descriptive and cost-effective explanation techniques for visualizing feature maps. Although the study showed that XAI could help select a desirable model depth for plant phenotypic tasks, using the framework in practice could be time-consuming. Domain expertise and understanding of the deep learning architecture might be required to perform the analysis and develop the desired model.

**Figure 5:**
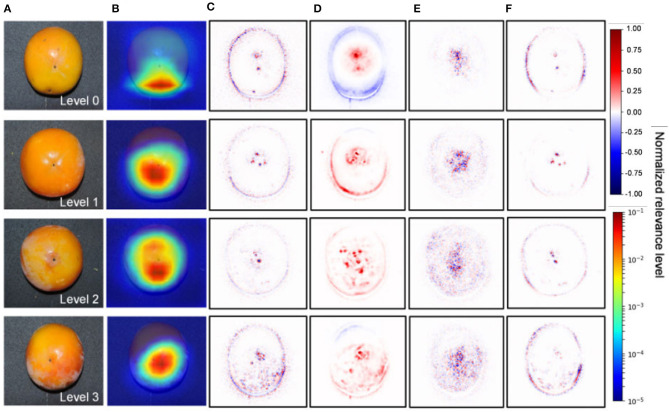
Visualization of the feature maps representing significant features contributing to the diagnosis of calyx-end cracking for the VGG16 model (Akagi et al., 2020). **(A)** Original image. **(B)** Grad-CAM. **(C)** Guided backpropagation. **(D)** LRP-Sequential B. **(E)** LRP-Epsilon. **(F)** Guided Grad-CAM.

The visualization of the feature maps of a deep learning model has helped plant scientists diagnose the internal disorder in persimmon fruit which the experts even missed. Akagi et al. (2020) developed a deep learning classifier capable of classifying calyx-end cracking in persimmon fruit. They used five different CNNs for the classification between healthy and cracking and achieved 90% accuracy. In the final step, the authors visualized the feature maps to detect cracking (Figure 5). The visualization showed higher relevance around the apex area and peripheral of the fruits, which might be related to particular stress. The lack of a large dataset to validate the findings is a significant drawback of the study.

The contribution of the appearance characteristics and the texture characteristics of leaf lesions during the feature extraction process of a deep learning model was studied by Wei et al. (2022). The authors trained VGG, GoogleNet, and ResNet for disease classification and used GradCAM, LIME, and Smoothgrad (Smilkov et al., 2017) to visualize the features learned by the models. The visualization showed that the pixels of the lesion position are the most important for the prediction. However, the authors were inconclusive of the contribution of the appearance of the leaves. During the comparison of the explanations generated by different XAI techniques, the authors found that the explanations generated by GradCAM were more intuitive and easy to understand than the Smoothgrad and LIME.

Mostafa et al. (2021, 2022) studied the relation between the depth of a deep learning model to its performance by using GBP. The authors proposed using a SSIM cut curve, which can help select the required depth of a model to achieve the desired performance by utilizing the structural similarity index (SSIM) of the feature maps generated at different depths of the model. In the study, different plant datasets were used to verify the results of the SSIM cut curve. Using the proposed algorithm the authors showed that higher depth deep learning models do not necessarily contribute to better performance.

Ghosal et al. (2017) proposed a classification model for foliar stresses in the soybean plant. The authors used GradCAM to isolate the visual symptoms that contribute to the model's prediction. Nagasubramanian et al. (2020) also proposed a DenseNet-121-based soybean stresses classifier and used several XAI techniques to understand the features learned by the model. The authors observed that sometimes the deep learning models learn features that might not be relevant to the infection in the plant.

In plant phenotyping, hyperspectral imaging (HSI) plays an important role. It allows for capturing plants' abiotic, biotic, chemical, and quality traits along with spatial and spectral information. In Nagasubramanian et al. (2019), the authors utilized HSI to develop a soybean disease classifier. They proposed a 3DCNN to utilize the HSI images' spectral and spatial information for the classification. Next, a saliency map (Simonyan et al., 2013) was used to detect the regions in the images that contributed to the prediction. Due to the use of HSI, the saliency maps helped detect the wavelength channel that maximally activated the feature maps. Using this information, a histogram showed the distribution of wavelengths across all the pixels. The use of saliency maps helped the authors authenticate the proposed method. This study opens new avenues of plant disease classification using HSI and deep learning.

Schramowski et al. (2020) proposed explanatory interactive learning, a framework for deep learning models in plant phenotyping. The authors used HSI images to demonstrate that inclusion of explanations of the decisions of a deep learning model into the model development process can help reveal Clever Hans (utilization of insignificant features within datasets) like behavior. The authors developed an interactive deep learning model where users can control the model development based on the explanations.

##### 3.2.1.2. Plant classification

Desai et al. (2019) proposed a classifier-based paddy rice's flowering panicle counter. Desai et al. (2019) used a sliding window that passes over the training image and extracts image patches. The patches were then used in a classifier to detect whether a flowering panicle was present in the patch. Depending on the presence of the flowering region, a bounding box is generated. Finally, the flowering regions are counted to get an estimation of the number of flowering in the image. The authors used GradCAM to observe the regions that the model used for classification and found that the flower regions mainly influenced the prediction.

A different approach for plant classification was adopted by Grinblat et al. (2016), where a vein morphological pattern was used to classify white bean, red bean, and soybean plants using deep learning models. The hit or miss algorithm by Soille (1999) was used to extract the veins and create a binary image. Next, patches of veins were cropped from the images, and the images were used to train a deep learning model, which was proposed in this study. In addition, the feature maps for different classes were visualized using the saliency map visualization by Zeiler and Fergus (2014). The visualization helped the authors realize that the model extracted features from different parts of veins for the prediction of different classes.

Minamikawa et al. (2022) proposed a method to automatically measure the morphological features of citrus fruits by the image analysis of cross-sectional images of citrus fruits. The authors used GradCAM to visualize the features in the fruit images that were important for the classification of peeling and fruit hardness. The authors combined GradCAM visualization with the information of the fruit morphological features to reveal key features important for the prediction. The authors proposed that it is important to connect the visualization results with knowledge on plant physiology and breeding to increase the reliability of deep learning models and understand the molecular mechanism of targeted traits.

#### 3.2.2. Regression

**Figure 6:**
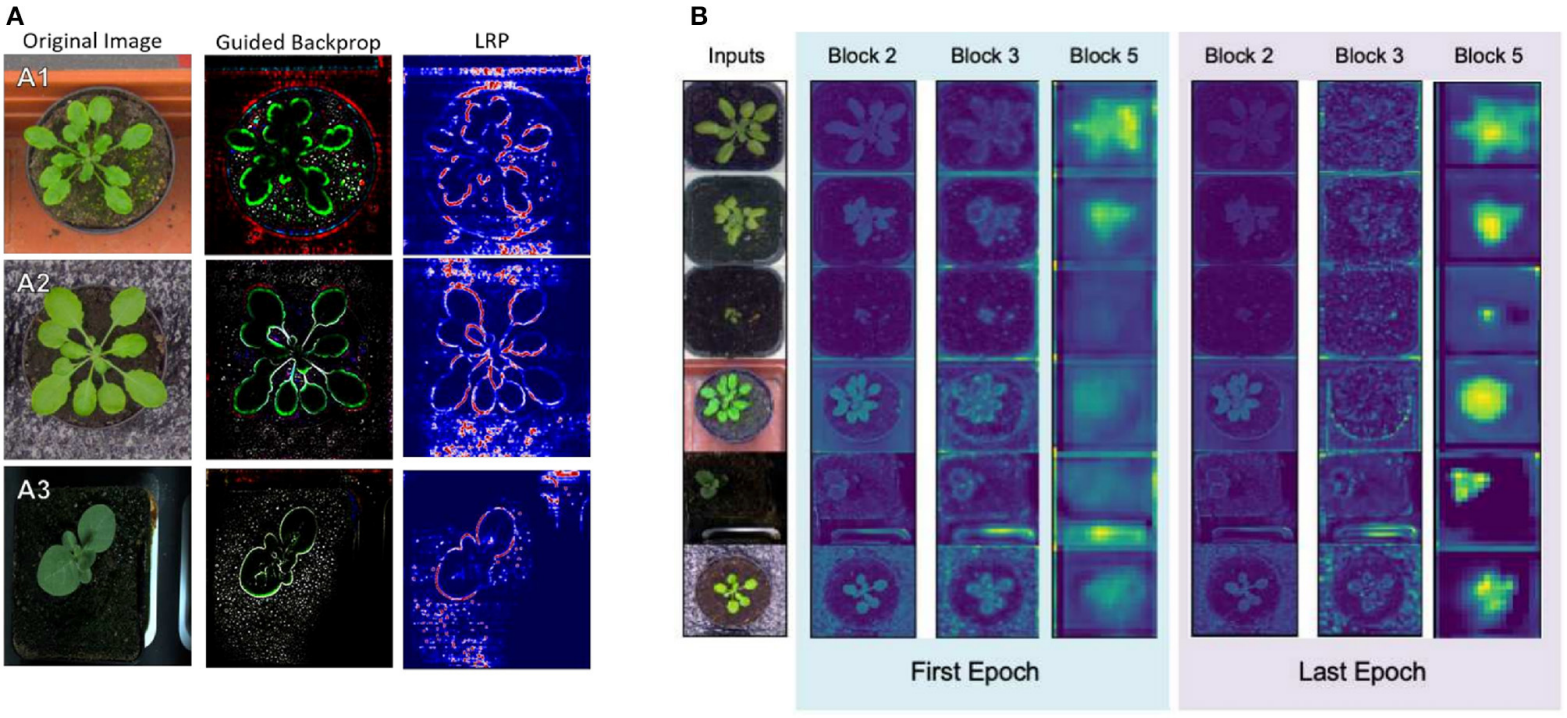
Visualization of the learning of a VGG16 based regression model using GBP and LRP (Dobrescu et al., 2019). **(A)** GBP and LRP visualization of different plants. **(B)** Average activations at the end of several convolutional blocks at the start and end of training.

In Dobrescu et al. (2017), the authors proposed a leaf counter for rosette plants. The salient regions that contributed to the regression were shown in work using a simple heatmap technique. It helped the authors emphasize that the model was learning from the leaf regions as obstructing the leaf generated error and was visible in the heatmap. To investigate the learning of the VGG16 based regression model counting plant leaves, Dobrescu et al. (2019) used GBP and LRP to visualize the feature maps of the model (Figure 6). The authors found that the initial layers learn low-level features, the deeper layers focus on the leaf edge, and the final layer produces the highest activation in the plant region. Experiments also showed that the regression model discards the leaf surface and uses the leaf edge information for counting. In a regression model, the visualization techniques may ensure that the model is indeed learning from the object of interest, allowing users to gain confidence in the results but also helps them explain the model accurately.

TasselNetV3 is a plant counting deep learning model that uses model explanation to enhance the human-level interpretability (Lu et al., 2021). In TasselNetV3, the authors proposed dynamic unfolding that assigns weights to the local region by learning from the ground-truth density maps. Dynamic unfolding replaces the averaging of the local count into the receptive field, which greatly improves the model performance. The authors also visualized the feature maps that helped them find which instances were counted. Such visualization helped to find what might have caused the model to fail.

Regression plays a crucial role in high-throughput plant phenotyping, facilitating rapid and precise counting of plants and plant organs (e.g., flowers, leaves, spikes, kernels). Through the XAI techniques, researchers have gained insight into the importance of object edges in regression and identified the probable cause for model failures. However, further investigation and validation across diverse datasets consisting of various imaging and field conditions are necessary to generalize and replicate these findings effectively.

#### 3.2.3. Synthetic data generation

Drees et al. (2022) proposed TransGrow, a conditional generative adversial network that can generate time-dependent high-quality and realistic images of irregular and incomplete sequences in above-ground plant phenotypes. TransGrow allows farmers to predict future above-ground phenotype at any time point in the growing season. In addition to proposing TransGrow, the authors visualized the pixel-wise uncertainty of plant growth for each time and found noticeable differences at the leaf edges, where the variance of plant growth is naturally the highest.

The lack of large plant phenotyping dataset has already been discussed. An overview of XAI studies in plant phenotyping is provided in Table 5.

**Table 5:** Overview of XAI studies in plant phenotyping.

**References**	**XAI technique**	**Purpose of XAI**	**Phenotyping task**	**Plant dataset**	**Model**
Ghosal et al. (2018)	Ranked features and generated saliency map of features	Explain model understanding	Identification of soybean stresses from plant leaves	Proposed dataset	Proposed model
Nagasubramanian et al. (2019)	Saliency map visualization	Track physiological insights of model prediction	Classification of charcoal rot	Proposed dataset	3D CNN
Toda and Okura (2019)	1. Occlusion analysis, 2. LIME, 3. GBP, 4. GradCAM, 5. DeepLIFT, 6. Explanation map	Interpret the representation of plant disease by a CNN	Plant disease classification	PlantVillage	InceptionV3
Grinblat et al. (2016)	Saliency map visualization	Understand the features learned by a CNN for classification	Plant classification using vain morphological pattern of white bean, red bean, and soybean	Larese et al. (2014)	Shallow CNN
Wei et al. (2022)	1. GradCAM, 2. LIME, 3. Smilkov et al. (2017)	Study the contribution of appearance and texture characteristics to model prediction	Leaf lesion classification	PlantVillage	1. VGG, 2. GoogleNet, 3. ResNet
Mostafa et al. (2021)	GBP	Selection of model depth and analyzing overfit model	Plant and leaf classification	1. PlantVillage	1. Shallow CNN
				2. Plant Seedling	2. ResNet50
				3. Beck et al., 2020	
Mostafa et al. (2022)	GBP	Selection of model depth	Plant and leaf classification	1. PlantVillage	1. Shallow CNN
				2. Plant Seedling	2. ResNet50
				3. Beck et al., 2020	
Ghosal et al. (2017)	GradCAM	Isolate visual symptoms that contribute to model prediction	Classification of foliar stresses in the soybean plant	PlantVillage	Proposed model
Nagasubramanian et al. (2020)	1. Saliency map, 2. SmoothGrad, 3. GBP. 4. Deep taylor decomposition, 5. Integrated gradients, 6. LRP, 7. Gradient times input	Compare different XAI techniques to interpret the prediction	Plant leaf classification	Ghosal et al., 2018	DenseNet-121
Minamikawa et al. (2022)	GradCAM	Visualize features relevant to the prediction	Measure the morphological features of citrus fruits	Proposed dataset	1. VGG16 2. ResNet50 3. InceptionV3 4. InceptionResNetv2
Akagi et al. (2020)	1. GradCAM, 2. GBP, 3. LRP, 4. Guided GradCAM, 5. InceptionResNetv2	Diagnose internal disorder in permission fruit using the visualization	Classify calyx-end cracking in persimmon fruit	Proposed dataset	1. AlexNet 2. VGG16 3. InceptionV3 4. ResNet50
Schramowski et al. (2020)	1. GradCAM	Analyze Clever Hans-like behavior in deep learning models	HSI classification	1. Proposed dataset	Proposed model
	2. LIME			2. Fashion MNIST	
				3. Pascal VOC 2007	
Desai et al. (2019)	GradCAM	Study image features that contribute toward the classification	Paddy rice's flowering panicle counter	Developed dataset	ResNet50
Dobrescu et al. (2019)	1. GBP	Study of the features extracted in regression	Count leaf of rosette plants	Leaf counting challenge Tsaftaris and Scharr (2017)	VGG16
	2. LRP				
Lu et al. (2021)	Proposed visualization technique	Human interpretable visualization of the learned features of the proposed model	Count maize tassels, wheat ears, and rice plants	1. Lu et al., 2017	Proposed model
				2. Madec et al., 2019	
				3. Liu et al., 2020	
Drees et al. (2022)	Proposed visualization	Data augmentation	Data augmentation	Proposed dataset	Proposed model

## 4. Significance of deep learning in plant science and future work for XAI

As the field of plant sciences continues to embrace the potential of deep learning, it is essential to recognize the ethical implications and biases that can arise in the development and use of these deep learning models. These biases can impact various aspects, including data collection, analysis, decision-making, and the overall outcomes of plant research. In this section, we explore the potential benefits of using deep learning, the challenges associated with it, and how explainability can help in addressing some of these challenges in adopting deep learning.

### 4.1. Significance of deep learning in plant science

Deep learning algorithms excel at analyzing large scale datasets and finding patterns within these datasets to gain insights that might not be feasible with traditional approaches to analyzing data. This can significantly reduce the amount of time required in decision-making processes and potentially uncover hidden insights that were previously thought not feasible to identify. Deep learning models can help in predictions for improving crop varieties by optimizing crops for disease and drought resistance, mineral nutrient uptake and planning to adapt to and also mitigate environmental change. By harnessing the power of deep learning algorithms, researchers can improve crops to be more resilient to environmental challenges and for better resource use efficiency.

Image-based analysis can be deployed in multiple scenarios to assess and enhance the performance of the plants in different conditions. For example, in disease and pest detection, images can be used to categorize and quantitatively assess diseases on a plant. In plant phenotyping to improve shoot and root architecture traits using plant breeding programs, deep learning algorithms can be used to improve plant image quality and data consistency while reducing the workload of researchers so they can focus on other important research components. Traditional image processing algorithms can be rigid to specific applications, while deep learning algorithms can model to provide generalization capability for processing images that aid plant phenotyping research. Employing deep learning for analyzing plant phenotyping datasets can significantly improve the throughput of the research as there is less time required to design tools specifically for all different applications. Hence, the application of deep learning to process images (plant phenotyping) related to changes in DNA sequence (plant genotyping) can help in accelerating breeding programs, ultimately leading to reduced time in generating improved varieties of plants.

Moreover, deploying deep learning models for analysis can help uncover insights that may have been thought too difficult to achieve before. For example, in plant research, the use of deep learning models can help uncover a phenotype in an abstract domain (such as latent space) that may help understand treatment effects on genetically different plants of the same species. In another example, a functional phenotype of root architecture can help understand variations in root architecture by comparing how similar they are to each other.

### 4.2. Challenges of deploying deep learning in plant research

The adoption of deep learning in plant science is impacted by many challenges associated with understanding how these models operate and provide insights into collected data. Moreover, it is critical to fully incorporate plant biologists who are experts in the specific area of research the deep learning model is being applied to, as plant growth is a complex process with a number of factors involved. Specifically, understanding Genotype by Environment (GxE) interaction to produce a phenotype is an active area of research. Plants have evolved elegant response systems to adapt to changes in their local environment. One example, in the soil, there can be a very complex heterogeneity of the concentrations of specific soil mineral nutrients the plant roots must absorb to thrive [especially the major fertilizer nutrients, nitrogen (*N*), phosphorous (*P*), and potassium (*K*)]. For example, the primary form of nitrogen absorbed from the spoil is the nitrate (*NO*_3_) anion. Nitrate is very mobile in soils and can move with the groundwater. Hence there can be patches of soil that are low or high in nitrate. Plant roots have evolved an elegant nitrate sensing and response system. This involves complex gene and protein networks that note when the soil nitrate is low and shut down lateral root growth. This enables more of the root carbon to be used by the primary root to grow faster and find regions of high soil nitrate. When these regions are accessed by the primary root, these networks then turn on and stimulate lateral root growth to absorb this much-needed nutrient (Remans et al., 2006; Wang Y.-Y. et al., 2018; Maghiaoui et al., 2020). Hence, relatively minor changes in the plant environment or plant genetic makeup could produce quite different plant phenotypes, be they above or below ground.

#### 4.2.1. Interpretation results derived by deep learning

As plant researchers embrace advances in deep learning to aid in enhancing crops for sustainable production of food, the relatively black box nature of the deep learning algorithms can deter some plant researchers from adopting these tools and techniques in their research programs.

Due to a lack of available and accessible data, some of the trained deep learning models augment datasets with simulated plant datasets. The field of plant research is always evolving, and it is difficult to model plant growth to simulate plant images. Some available tools for simulating plants digitally do not consider all the different factors involved in plant growth. For example, a tool developed by (OpenSimRoot), simulates plant root growth based on multiple parameters. However, it assumes that when drought-stressed, bean roots tend to grow deeper, which might not be completely true. Drought often quickly inhibits both root and shoot growth (Reinelt et al., 2023). When a deep learning model is trained with such simulated datasets, it raises the question of whether the bias of the simulator is built into the deep learning model as well and may prevent other researchers from using it. In a different scenario, if it was used by a research group, the built-in bias would propagate through other research projects and lead to improper outcomes.

Abstract phenotypes obtained by using machine learning models proposed by Ubbens et al. (2020), (based on latent space phenotyping) may not help plant scientists understand what that means even after finding a region of interest other than that there is some difference in latent space phenotype for treated vs non-treated plants of the same line. Being able to interpret what that actually means in the realms of plant physiology may be more useful to other researchers. There is a huge potential for new phenotypes, such as latent space phenotype, in understanding plant function, but having a translation of what that actually means in real-world physiology is as important.

#### 4.2.2. Biased dataset

The deep learning models are trained with large datasets of images or other data related to plant phenotyping. These trained models can inherit the bias present in the dataset itself. For example, training a model with an image dataset that consists of data points collected in highly controlled environments such as growth chambers with highly regulated lighting and temperature growth conditions can make it biased toward predicting better with images acquired in those conditions, but could fail to predict when the deep learning algorithm is subjected to images from real-world conditions where light and temperature (and other conditions) can vary significantly throughout the day/night cycle. From our experience, for phenotyping plant roots grown in hydroponics, even in the more controlled growth chambers, root growth can be affected by a number of factors, including changes in temperatures, light and humidity in different growth chambers, use of different materials for germination, the *pH* of nutrient solutions, and proper aeration within the growth solution. When imaging the plant root systems, factors that can impact datasets, which may not be directly related to genetic variation, include proper lighting conditions, the color of the filter paper used to provide optimal contrast with the white roots, imaging roots placed under water vs out of the water, camera parameters such as exposure, aperture and shutter speed, and presence of a meniscus along roots that are grown in water but imaged in air, can lead to generating biased datasets that can result in deep learning model favoring one imaging condition than other.

These biased datasets raise concerns about the generalizability and reliability of deep learning models and can lead to a skeptical view about the efficacy of these approaches by some plant researchers. Another possibility is that the use of these models as de facto state-of-the-art models in assessing plant varieties can result in poor decision-making and skewed, possibly wrong, results being published in high-quality journals, which are followed by other researchers. This can have a negative ripple effect on the future of plant research.

#### 4.2.3. Ethical considerations

Incorporating ethical considerations while training large-scale deep learning models for use in plant science can help ensure reliability and impact on the agricultural environment and stakeholders, including farmers, breeders, and consumers. For instance, models trained on biased datasets from one region and plant species or genetic variety could lead to challenges in another region, such as improper use of resources such as water and environmental degradation, for example, from overuse of fertilizers. Another example could be where a deep learning model trained with a dataset acquired from a highly controlled environment being deployed in a field breeding program or farm, resulting in crop failures due to the lack of ground truth data from the lab with plant growth in the real world, the farmer's field. This points to a lack of accountability in training such models.

Transparency in the availability of datasets used to train such models could be another aspect that can be put under the lens. In today's competitive research environment, focus on publishing results of perceived “black box” deep learning models rather than on open access and transparency in availing datasets and other experimental materials (including protocols and growth conditions) could be potentially more harmful to plant research than benefiting. Researchers should strive for transparency by openly sharing methodologies, data sources, and model architectures. This enables the scientific community to scrutinize and evaluate the reliability, biases, and potential limitations of deep learning models. Considerations should be given to the potential unintended consequences of altering plant root architectures, such as potential ecological disruptions or unforeseen impacts on soil health or nutrient cycling. Furthermore, focusing solely on agricultural productivity without considering ecological sustainability can lead to detrimental consequences, such as soil erosion, loss of biodiversity, and negative impacts on ecosystems.

### 4.3. Impact of explainability and interpretability

Explainability can provide transparency and build trust among researchers, policymakers, and stakeholders in the plant science community. When deep learning models are interpretable, researchers can understand how the models make predictions or decisions. This transparency helps to mitigate skepticism or reluctance surrounding the adoption of deep learning, ensuring that stakeholders have confidence in the reliability and accuracy of the deep learning model-driven solutions.

XAI allows researchers to gain scientific insights and validate the results obtained through deep learning models. By understanding the factors or features that contribute to specific predictions or outcomes, researchers can validate whether the deep learning models align with existing scientific knowledge or identify novel insights. This promotes robust scientific inquiry and ensures that deep learning is used as a tool to augment research rather than replace traditional scientific methods. XAI aids in identifying biases or unintended consequences in deep learning models. Researchers can examine the underlying data, algorithms, and decision-making processes to uncover biases or discriminatory patterns. This insight enables researchers to rectify biases, promote fairness, and ensure that deep learning models do not perpetuate inequitable practices in plant science research. XAI also facilitates collaboration among researchers by enabling them to share and discuss deep learning models, methodologies, and findings. Interpretable deep learning models provide a common ground for researchers to analyze, critique, and improve upon each other's work. This collaborative environment fosters collective learning, encourages interdisciplinary approaches, and advances the adoption of deep learning in plant science research. XAI can also help in knowledge transfer and education about deep learning in plant science research. Researchers can explain the workings of deep learning models to non-experts, policymakers, or the general public in a comprehensible manner. This fosters a broader understanding of deep learning and its applications, dispelling misconceptions or fears surrounding the technology. By promoting deep learning literacy, explainability paves the way for wider adoption and acceptance of deep learning in plant science research.

In conclusion, XAI can play a pivotal role in the adoption of deep learning in plant research by fostering trust, enabling scientific validation, being a more inclusive environment for plant scientists, identifying biases, facilitating collaboration, ensuring compliance with regulations and ethical guidelines, and promoting deep learning literacy. By prioritizing XAI, researchers can effectively harness the benefits of deep learning while addressing concerns and promoting the responsible and transparent use of deep learning in plant science research.

## 5. Proposal for an XAI framework in plant phenotyping

**Figure 7:**
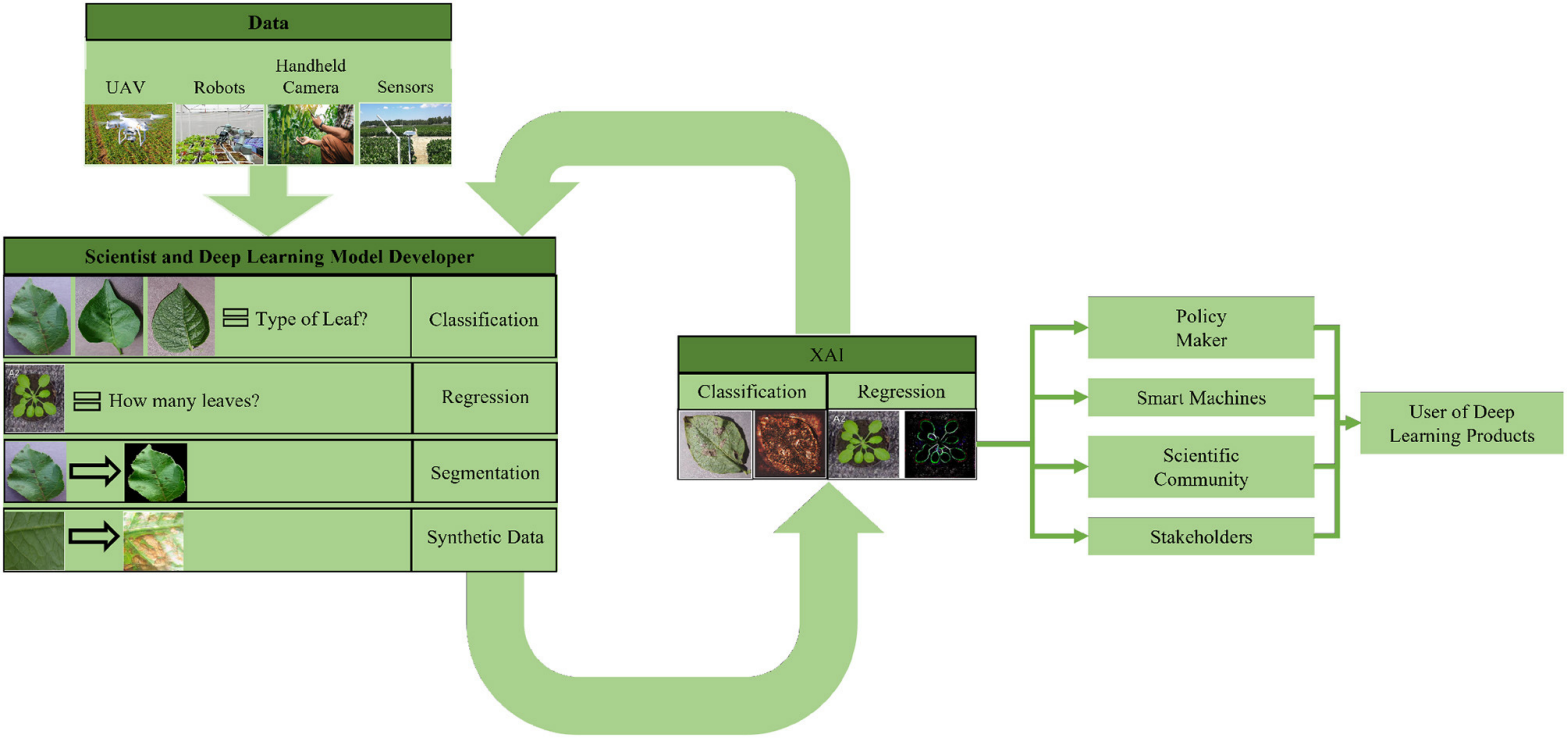
Overview of a framework incorporating XAI in deep learning based plant phenotyping studies.

Adaptation of XAI techniques is still in the early stages for plant phenotyping studies. In this section, we propose an XAI framework that can help researchers understand the steps required to utilize XAI techniques. An overview of the proposed framework is illustrated in Figure 7. The first step of the framework is the collection of data. The recent advancements in smart machines, cameras, and sensors have helped us acquire large amounts of plant phenotyping data. The data is directly collected from the fields. The next step is the utilization of the data for developing deep learning models performing plant phenotyping tasks. Plant and computer scientists, biologists, agriculturalists, and researchers in the industry are using the data to develop deep learning models which are performing different plant phenotyping tasks, e.g., classification of plants and diseases associated with plants, counting of different plant parts, segmentation of specific plant parts, and generation of synthetic plant phenotyping data. Although scientists are using deep learning models to achieve superior results, the black-box nature of these models means that the scientists may not fully understand the model's behavior. So, the next step is the integration of XAI techniques. Researchers can use XAI techniques to understand the performance of a model, which can help them improve the performance of a model. Additionally, researchers can take advantage of the XAI techniques to explain the results of a model, providing users with an added level of transparency and reliability.

Data scientists can benefit from XAI by understanding the features responsible for a model's decision, while plant scientists, biologists, and agriculturalists can find the plant traits responsible for the model's superior performance. A proper understanding of a model's decision will help increase trust in deep learning models, and in turn, inform policies and regulations regarding their deployment in safety critical sectors, such as food production. User trust is also important for stakeholders in safety critical sectors. XAI can also help practitioners take the necessary steps to regularize and standardize deep learning studies. XAI can help explain whether the data is used properly to develop a deep learning model, and most importantly, whether the deep learning model is making the correct decisions. In the development of smart machines, XAI can help greatly. As smart machines are mostly automated and have to make decisions on their own, XAI can help developers find whether the deep learning model is considering relevant and desired features to make the decisions.

## 6. Conclusion

In this study, we conducted a comprehensive analysis of XAI techniques used in deep learning studies in the context of plant phenotyping research. Our study revealed that deep learning models have the potential to uncover novel plant traits and provide more efficient and accurate tools for plant phenotyping. Additionally, we discovered that XAI techniques can elucidate the outcomes of deep learning models and present avenues for enhancing existing models. We identified areas where XAI has potential across the various task domains of plant phenotyping. Although the existing XAI literature predominantly focuses on classification models, leveraging XAI techniques in other deep learning models (e.g., regression, generative) could propel advancements in plant phenotyping. Consequently, our review serves as a valuable reference for integrating deep learning models into plant data analysis and underscores the significance of XAI in such investigations.

## Author contributions

SM and DM conceived the idea for the review paper. SM conducted the literature review and compiled the data. DM and IS supervised the research. SM, DM, and IS prepared the initial manuscript. KP and LK introduced root phenotyping in the discussion, included the perspective of plant scientists in the study, and provided critical feedback. All authors contributed to the writing of the manuscript and approved it for publication.

## References

[B1] Adadi A. Berrada M. (2018). Peeking inside the black-box: a survey on explainable artificial intelligence (XAI). IEEE Access 6, 52138–52160. 10.1109/ACCESS.2018.2870052

[B2] Adebayo J. Gilmer J. Goodfellow I. Kim B. (2018a). Local explanation methods for deep neural networks lack sensitivity to parameter values. arXiv:1810.03307v1.

[B3] Adebayo J. Gilmer J. Muelly M. Goodfellow I. Hardt M. Kim B. (2018b). Sanity checks for saliency maps. Adv. Neural Inf. Process. Syst. 31.

[B4] Agarwal R. Melnick L. Frosst N. Zhang X. Lengerich B. Caruana R.. (2021). Neural additive models: interpretable machine learning with neural nets. Adv. Neural Inf. Process. Syst. 34, 4699–4711. Available online at: https://proceedings.neurips.cc/paper_files/paper/2021/file/251bd0442dfcc53b5a761e050f8022b8-Paper.pdf36850392

[B5] Aich S. Stavness I. (2017). “Leaf counting with deep convolutional and deconvolutional networks,” in Proceedings of the IEEE International Conference on Computer Vision Workshops (Italy), 2080–2089.

[B6] Akagi T. Onishi M. Masuda K. Kuroki R. Baba K. Takeshita K.. (2020). Explainable deep learning reproduces a 'professional eye” on the diagnosis of internal disorders in persimmon fruit. Plant Cell Physiol. 61, 1967–1973. 10.1093/pcp/pcaa111 32845307

[B7] Almahairi A. Rajeshwar S. Sordoni A. Bachman P. Courville A. (2018). “Augmented cyclegan: learning many-to-many mappings from unpaired data,” in International Conference on Machine Learning (Sweden: PMLR), 195–204.

[B8] Alvarez Melis D. Jaakkola T. (2018). Towards robust interpretability with self-explaining neural networks. Adv. Neural Inf. Process. Syst. 31.

[B9] Amara J. Bouaziz B. Algergawy A. (2017). “A deep learning-based approach for banana leaf diseases classification,” in Datenbanksysteme für Business, Technologie und Web (BTW 2017)-Workshopband (Bonn: Gesellschaft für Informatik).

[B10] Ampatzidis Y. Partel V. (2019). UAV-based high throughput phenotyping in citrus utilizing multispectral imaging and artificial intelligence. Remote Sens. 11, 410. 10.3390/rs11040410

[B11] Ancona M. Ceolini E. Öztireli C. Gross M. (2017). Towards better understanding of gradient-based attribution methods for deep neural networks. arXiv:1711.06104v4.

[B12] Angelov P. Soares E. (2020). Towards explainable deep neural networks (xDNN). Neural Netw. 130, 185–194. 10.1016/j.neunet.2020.07.010 32682084

[B13] Arras L. Horn F. Montavon G. Müller K.-R. Samek W. (2016). Explaining predictions of non-linear classifiers in NLP. arXiv. 10.18653/v1/W16-1601

[B14] Arras L. Horn F. Montavon G. Müller K.-R. Samek W. (2017). “What is relevant in a text document?”: an interpretable machine learning approach. PLoS ONE 12, e0181142. 10.1371/journal.pone.0181142 28800619 PMC5553725

[B15] Arrieta A. B. Díaz-Rodríguez N. Del Ser J. Bennetot A. Tabik S. Barbado A.. (2020). Explainable artificial intelligence (XAI): concepts, taxonomies, opportunities and challenges toward responsible AI. Inf. Fus. 58, 82–115. 10.1016/j.inffus.2019.12.012

[B16] Arya S. Sandhu K. S. Singh J. Kumar S. (2022). Deep learning: as the new frontier in high-throughput plant phenotyping. Euphytica 218, 1–22. 10.1007/s10681-022-02992-3

[B17] Askew K. (2017). Population Growth “a Threat to Food Quality.” Available online at: https://www.foodnavigator.com/Article/2017/11/10/Population\discretionarygrowth-a-threat-to-food-quality#:\protect\kern+.2222em\relaxtext=Global%20population%20growth%20means%20that,reaches%209.1bn%20by%202050 (accessed January 21, 2023).

[B18] Assaf R. Schumann A. (2019). “Explainable deep neural networks for multivariate time series predictions,” in IJCAI (Macao), 6488–6490.

[B19] Atkinson J. A. Pound M. P. Bennett M. J. Wells D. M. (2019). Uncovering the hidden half of plants using new advances in root phenotyping. Curr. Opin. Biotechnol. 55, 1–8. 10.1016/j.copbio.2018.06.002 30031961 PMC6378649

[B20] Bach S. Binder A. Montavon G. Klauschen F. Müller K.-R. Samek W. (2015). On pixel-wise explanations for non-linear classifier decisions by layer-wise relevance propagation. PLoS ONE 10, e0130140. 10.1371/journal.pone.0130140 26161953 PMC4498753

[B21] Badrinarayanan V. Kendall A. Cipolla R. (2017). Segnet: a deep convolutional encoder-decoder architecture for image segmentation. IEEE Trans. Pattern Anal. Mach. Intell. 39, 2481–2495. 10.1109/TPAMI.2016.2644615 28060704

[B22] Bai X. Wang X. Liu X. Liu Q. Song J. Sebe N.. (2021). Explainable deep learning for efficient and robust pattern recognition: a survey of recent developments. Pattern Recognit. 120, 108102. 10.1016/j.patcog.2021.108102

[B23] Bauer A. Bostrom A. G. Ball J. Applegate C. Cheng T. Laycock S.. (2019). Combining computer vision and deep learning to enable ultra-scale aerial phenotyping and precision agriculture: a case study of lettuce production. Hortic. Res. 6. 10.1038/s41438-019-0151-5 31231528 PMC6544649

[B24] Beck M. A. Liu C.-Y. Bidinosti C. P. Henry C. J. Godee C. M. Ajmani M. (2020). An embedded system for the automated generation of labeled plant images to enable machine learning applications in agriculture. PLoS ONE 15, e0243923. 10.1371/journal.pone.0243923 33332382 PMC7745972

[B25] Belle V. Papantonis I. (2021). Principles and practice of explainable machine learning. Front. Big Data 39, 688969. 10.3389/fdata.2021.688969 34278297 PMC8281957

[B26] Bellucci M. Delestre N. Malandain N. Zanni-Merk C. (2021). Towards a terminology for a fully contextualized XAI. Proc. Comput. Sci. 192, 241–250. 10.1016/j.procs.2021.08.025

[B27] Berg A. Deng J. Fei-Fei L. (2010). Large Scale Visual Recognition Challenge (ILSVRC, 2). Available online at: https://www.image-net.org/challenges/LSVRC/ (accessed December 05, 2022).

[B28] Bernotas G. Scorza L. C. Hansen M. F. Hales I. J. Halliday K. J. Smith L. N.. (2019). A photometric stereo-based 3D imaging system using computer vision and deep learning for tracking plant growth. GigaScience 8, giz056. 10.1093/gigascience/giz056 31127811 PMC6534809

[B29] Bhatt U. Xiang A. Sharma S. Weller A. Taly A. Jia Y.. (2020). “Explainable machine learning in deployment,” in Proceedings of the 2020 Conference on Fairness, Accountability, and Transparency (Barcelona), 648–657.

[B30] Biran O. Cotton C. (2017). “Explanation and justification in machine learning: a survey,” in IJCAI-17 Workshop on Explainable AI (XAI) (Melbourne), *Vol. 8*, 8–13.

[B31] Blitzer J. Dredze M. Pereira F. (2007). “Biographies, bollywood, boom-boxes and blenders: domain adaptation for sentiment classification,” in Proceedings of the 45th Annual Meeting of the Association of Computational Linguistics (Prague), 440–447.

[B32] Brunese L. Mercaldo F. Reginelli A. Santone A. (2020). Explainable deep learning for pulmonary disease and coronavirus COVID-19 detection from X-rays. Comput. Methods Progr. Biomed. 196, 105608. 10.1016/j.cmpb.2020.105608 32599338 PMC7831868

[B33] Burns C. Thomason J. Tansey W. (2020). “Interpreting black box models via hypothesis testing,” in Proceedings of the 2020 ACM-IMS on Foundations of Data Science Conference, 47–57.30808574

[B34] Cabitza F. Rasoini R. Gensini G. F. (2017). Unintended consequences of machine learning in medicine. JAMA 318, 517–518. 10.1001/jama.2017.7797 28727867

[B35] Cap Q. H. Uga H. Kagiwada S. Iyatomi H. (2022). LeafGAN: an effective data augmentation method for practical plant disease diagnosis. IEEE Trans. Autom. Sci. Eng. 19, 1258–1267. 10.1109/TASE.2020.3041499

[B36] Caruana R. Lou Y. Gehrke J. Koch P. Sturm M. Elhadad N. (2015). “Intelligible models for healthcare: predicting pneumonia risk and hospital 30-day readmission,” in Proceedings of the 21th ACM SIGKDD International Conference on Knowledge Discovery and Data Mining (Sydney), 1721–1730.

[B37] Chandra A. L. Desai S. V. Guo W. Balasubramanian V. N. (2020). Computer vision with deep learning for plant phenotyping in agriculture: a survey. *arXiv*. Available online at: https://arxiv.org/abs/2006.11391v1

[B38] Chattopadhay A. Sarkar A. Howlader P. Balasubramanian V. N. (2018). “Grad-cam++: generalized gradient-based visual explanations for deep convolutional networks,” in 2018 IEEE Winter Conference on Applications of Computer Vision (WACV) (Lake Tahoe, NV: IEEE), 839–847.

[B39] Chatzimparmpas A. Martins R. M. Jusufi I. Kerren A. (2020). A survey of surveys on the use of visualization for interpreting machine learning models. Inf. Vis. 19, 207–233. 10.1177/1473871620904671

[B40] Che Z. Purushotham S. Khemani R. Liu Y. (2015). Distilling knowledge from deep networks with applications to healthcare domain. arXiv:1512.03542v1.

[B41] Chen H. Engkvist O. Wang Y. Olivecrona M. Blaschke T. (2018). The rise of deep learning in drug discovery. Drug Discov. Today 23, 1241–1250. 10.1016/j.drudis.2018.01.039 29366762

[B42] Chouhan S. S. Singh U. P. Kaul A. Jain S. (2019). “A data repository of leaf images: practice towards plant conservation with plant pathology,” in 2019 4th International Conference on Information Systems and Computer Networks (ISCON) (India: IEEE), 700–707.

[B43] Cohen J. P. Morrison P. Dao L. (2020). Covid-19 image data collection. arXiv:2003.11597v1.

[B44] Danilevicz M. F. Gill M. Anderson R. Batley J. Bennamoun M. Bayer P. E.. (2022). Plant genotype to phenotype prediction using machine learning. Front. Genet. 13, 822173. 10.3389/fgene.2022.822173 35664329 PMC9159391

[B45] Danilevsky M. Qian K. Aharonov R. Katsis Y. Kawas B. Sen P. (2020). A survey of the state of explainable ai for natural language processing. arXiv:2010.00711v1.

[B46] Das A. Rad P. (2020). Opportunities and challenges in explainable artificial intelligence (XAI): a survey. arXiv:2006.11371v2.

[B47] DeChant C. Wiesner-Hanks T. Chen S. Stewart E. L. Yosinski J. Gore M. A.. (2017). Automated identification of northern leaf blight-infected maize plants from field imagery using deep learning. Phytopathology 107, 1426–1432. 10.1094/PHYTO-11-16-0417-R 28653579

[B48] Deng J. Dong W. Socher R. Li L.-J. Li K. Fei-Fei L. (2009). “ImageNet: a large-scale hierarchical image database,” in IEEE Conference on Computer Vision and Pattern Recognition, 248–255. 10.1109/CVPR.2009.520684826886976

[B49] Deng L. (2012). The mnist database of handwritten digit images for machine learning research. IEEE Signal Process. Mag. 29, 141–142. 10.1109/MSP.2012.2211477

[B50] Desai S. V. Balasubramanian V. N. Fukatsu T. Ninomiya S. Guo W. (2019). Automatic estimation of heading date of paddy rice using deep learning. Plant Methods 15, 1–11. 10.1186/s13007-019-0457-1 31338116 PMC6626381

[B51] Dignum V. (2017). Responsible artificial intelligence: designing AI for human values. ICT Discov. 1, 18. Available online at: https://www.itu.int/dms_pub/itu-s/opb/journal/S-JOURNAL-ICTF.VOL1-2018-1-P01-PDF-E.pdf

[B52] Dobos O. Horvath P. Nagy F. Danka T. Viczián A. (2019). A deep learning-based approach for high-throughput hypocotyl phenotyping. Plant Physiol. 181, 1415–1424. 10.1104/pp.19.00728 31636105 PMC6878028

[B53] Dobrescu A. Valerio Giuffrida M. Tsaftaris S. A. (2017). “Leveraging multiple datasets for deep leaf counting,” in Proceedings of the IEEE International Conference on Computer Vision Workshops, 2072–2079.32256503

[B54] Dobrescu A. Valerio Giuffrida M. Tsaftaris S. A. (2019). “Understanding deep neural networks for regression in leaf counting,” in Proceedings of the IEEE/CVF Conference on Computer Vision and Pattern Recognition Workshops (Long Beach, CA).

[B55] Drees L. Weber I. Rußwurm M. Roscher R. (2022). “Time dependent image generation of plants from incomplete sequences with cnn-transformer,” in DAGM German Conference on Pattern Recognition (Konstanz: Springer), 495–510.

[B56] Dua D. Graff C.. (2017). UCI Machine Learning Repository (Irvine, CA).

[B57] Ellis K. A. Bush A. I Darby D. Fazio D. D. Foster J. Hudson P. (2009). The australian imaging, biomarkers and lifestyle (AIBL) study of aging: methodology and baseline characteristics of 1112 individuals recruited for a longitudinal study of Alzheimer's disease. Int. psychogeriatr. 21, 672–687. 10.1017/S1041610209009405 19470201

[B58] Erion G. Janizek J. D. Sturmfels P. Lundberg S. Lee S. I. (2019) Learning explainable models using attribution priors. arXiv preprint arXiv:1906.10670.

[B59] Esteva A. Robicquet A. Ramsundar B. Kuleshov V. DePristo M. Chou K.. (2019). A guide to deep learning in healthcare. Nat. Med. 25, 24–29. 10.1038/s41591-018-0316-z 30617335

[B60] EU (2018). Population Growth “a Threat to Food Quality.” Available online at: https://eur-lex.europa.eu/legal-content/EN/TXT/HTML/?uri=OJ:L:2016:119:FULL (accessed November 11, 2022).

[B61] Everingham M. Eslami S. Van Gool L. Williams C. K. Winn J. Zisserman A. (2015). The pascal visual object classes challenge: a retrospective. Int. J. Comput. Vis. 111, 98–136. 10.1007/s11263-014-0733-5

[B62] Everingham M. Winn J. (2011). The PASCAL visual object classes challenge 2012 (VOC2012) development kit. Pattern Anal. Stat. Model. Comp. Learn. Tech. Rep. 8, 5. Available online at: http://host.robots.ox.ac.uk/pascal/VOC/voc2012/devkit_doc.pdf

[B63] Falk K. G. Jubery T. Z. Mirnezami S. V. Parmley K. A. Sarkar S. Singh A.. (2020). Computer vision and machine learning enabled soybean root phenotyping pipeline. Plant Methods 16, 1–19. 10.1186/s13007-019-0550-5 31993072 PMC6977263

[B64] Fei-Fei L. Fergus R. Perona P. (2006). One-shot learning of object categories. IEEE Trans. Pattern Anal. Mach. Intell. 28, 594–611. 10.1109/TPAMI.2006.79 16566508

[B65] Feldman M. J. Ellsworth P. Z. Fahlgren N. Gehan M. A. Cousins A. B. Baxter I. (2018). Components of water use efficiency have unique genetic signatures in the model C4 Grass Setaria. Plant Physiol. 178, 699–715. 10.1104/pp.18.00146 30093527 PMC6181048

[B66] Fukushima K. (1988). Neocognitron: a hierarchical neural network capable of visual pattern recognition. Neural Netw. 1, 119–130. 10.1016/0893-6080(88)90014-721482455

[B67] Gaggion N. Ariel F. Daric V. Lambert E. Legendre S. Roulé T.. (2021). Chronoroot: high-throughput phenotyping by deep segmentation networks reveals novel temporal parameters of plant root system architecture. GigaScience 10, giab052. 10.1101/2020.10.27.350553 34282452 PMC8290196

[B68] Gebbers R. Adamchuk V. I. (2010). Precision agriculture and food security. Science 327, 828–831. 10.1126/science.1183899 20150492

[B69] Gerlings J. Shollo A. Constantiou I. (2020). Reviewing the need for explainable artificial intelligence (XAI). arXiv. 10.24251/HICSS.2021.15633079674

[B70] Ghorbani A. Abid A. Zou J. (2019a). Interpretation of neural networks is fragile. Proc. AAAI Conf. Artif. Intell. 33, 3681–3688. 10.1609/aaai.v33i01.33013681

[B71] Ghorbani A. Wexler J. Zou J. Y. Kim B. (2019b). Towards automatic concept-based explanations. Adv. Neural Inf. Process. Syst. (Vancouver), 32.

[B72] Ghosal S. Blystone D. Singh A. K. Ganapathysubramanian B. Singh A. Sarkar S. (2017). Interpretable deep learning applied to plant stress phenotyping. arXiv:1710.08619v3.

[B73] Ghosal S. Blystone D. Singh A. K. Ganapathysubramanian B. Singh A. Sarkar S. (2018). An explainable deep machine vision framework for plant stress phenotyping. Proc. Nat. Acad. Sci. U. S. A. 115, 4613–4618. 10.1073/pnas.1716999115 29666265 PMC5939070

[B74] Ghosal S. Zheng B. Chapman S. C. Potgieter A. B. Jordan D. R. Wang X.. (2019). A weakly supervised deep learning framework for sorghum head detection and counting. Plant Phenom. 2019. 10.34133/2019/1525874 33313521 PMC7706102

[B75] Gilpin L. H. Bau D. Yuan B. Z. Bajwa A. Specter M. Kagal L. (2018). “Explaining explanations: an overview of interpretability of machine learning,” in 2018 IEEE 5th International Conference on Data Science and Advanced Analytics (DSAA) (Turin: IEEE), 80–89.

[B76] Godfray H. C. J. Beddington J. R. Crute I. R. Haddad L. Lawrence D. Muir J. F.. (2010). Food security: the challenge of feeding 9 billion people. Science 327, 812–818. 10.1126/science.1185383 20110467

[B77] Gong L. Du X. Zhu K. Lin C. Lin K. Wang T.. (2021). Pixel level segmentation of early-stage in-bag rice root for its architecture analysis. Comp. Electron. Agric. 186, 106197. 10.1016/j.compag.2021.106197

[B78] Goodfellow I. Pouget-Abadie J. Mirza M. Xu B. Warde-Farley D. Ozair S.. (2014). Generative adversarial nets. Adv. Neural Inform. Process. Syst. 2014, 2672–2680.

[B79] Griffin G. Holub A. Perona P. (2007). Caltech-256 Object Category Dataset. Technical Report 7694.36844695

[B80] Grinblat G. L. Uzal L. C. Larese M. G. Granitto P. M. (2016). Deep learning for plant identification using vein morphological patterns. Comp. Electron. Agric. 127, 418–424. 10.1016/j.compag.2016.07.003

[B81] Grosenick L. Greer S. Knutson B. (2008). Interpretable classifiers for fmri improve prediction of purchases. IEEE Transact. Neural Syst. Rehabil. Eng. 16, 539–548. 10.1109/TNSRE.2008.926701 19144586

[B82] Guidotti R. Monreale A. Ruggieri S. Turini F. Giannotti F. Pedreschi D. (2018). A survey of methods for explaining black box models. ACM Comp. Surv. 51, 1–42. 10.1145/3236009

[B83] Gulum M. A. Trombley C. M. Kantardzic M. (2021). A review of explainable deep learning cancer detection models in medical imaging. Appl. Sci. 11, 4573. 10.3390/app11104573

[B84] Gunning D. Vorm E. Wang J. Y. Turek M. (2021). Darpa's explainable AI (XAI) program: a retrospective. Appl. AI Lett. 2, e61. 10.1002/ail2.61

[B85] Guo W. Zheng B. Potgieter A. B. Diot J. Watanabe K. Noshita K.. (2018). Aerial imagery analysis-quantifying appearance and number of sorghum heads for applications in breeding and agronomy. Front. Plant Sci. 9, 1544. 10.3389/fpls.2018.01544 30405675 PMC6206408

[B86] Haralick R. M. Shapiro L. G. (1985). Image segmentation techniques. Comp. Vis. Grap. Image Process. 29, 100–132. 10.1016/S0734-189X(85)90153-7

[B87] Harfouche A. L. Nakhle F. Harfouche A. H. Sardella O. G. Dart E. Jacobson D. (2022). A primer on artificial intelligence in plant digital phenomics: embarking on the data to insights journey. Trends Plant Sci. 28, 154–184. 10.1016/j.tplants.2022.08.021 36167648

[B88] Hasan A. M. Sohel F. Diepeveen D. Laga H. Jones M. G. (2021). A survey of deep learning techniques for weed detection from images. Comp. Electron. Agric. 184, 106067. 10.1016/j.compag.2021.106067

[B89] Hati A. J. Singh R. R. (2021). Artificial intelligence in smart farms: plant phenotyping for species recognition and health condition identification using deep learning. AI 2, 274–289. 10.3390/ai2020017

[B90] He K. Gkioxari G. Dollár P. Girshick R. (2017). “Mask R-CNN,” in Proceedings of the IEEE International Conference on Computer Vision (Venice), 2961–2969.

[B91] Hendricks L. A. Akata Z. Rohrbach M. Donahue J. Schiele B. Darrell T. (2016). “Generating visual explanations,” in European Conference on Computer Vision (Amsterdam: Springer), 3–19.

[B92] Holzinger A. Carrington A. Müller H. (2020). Measuring the quality of explanations: the system causability scale (SCS). Künstliche Intell. 34, 193–198. 10.1007/s13218-020-00636-z 32549653 PMC7271052

[B93] Hu X. Chu L. Pei J. Liu W. Bian J. (2021). Model complexity of deep learning: a survey. Knowl. Inf. Syst. 63, 2585–2619. 10.1007/s10115-021-01605-0

[B94] Huang Q. Yamada M. Tian Y. Singh D. Chang Y. (2022). Graphlime: Local interpretable model explanations for graph neural networks. IEEE Trans. Knowl. Data Eng. 35, 6968–6972. 10.1109/TKDE.2022.3187455

[B95] Hughes D. Salathé M. (2015). An open access repository of images on plant health to enable the development of mobile disease diagnostics. arXiv:1511.08060v2.

[B96] Ibrahim M. Louie M. Modarres C. Paisley J. (2019). “Global explanations of neural networks: mapping the landscape of predictions,” in Proceedings of the 2019 AAAI/ACM Conference on AI, Ethics, and Society (Honolulu, HI), 279–287.

[B97] Ivakhnenko A. G. (1968). The group method of data handling, a rival of the method of stochastic approximation. Soviet Automat. Control 13, 43–55.

[B98] Janiesch C. Zschech P. Heinrich K. (2021). Machine learning and deep learning. Electron. Mark. 31, 685–695. 10.1007/s12525-021-00475-2

[B99] Jiang Y. Li C. (2020). Convolutional neural networks for image-based high-throughput plant phenotyping: a review. Plant Phenom. 2020. 10.34133/2020/4152816 33313554 PMC7706326

[B100] Jiménez-Luna J. Grisoni F. Schneider G. (2020). Drug discovery with explainable artificial intelligence. Nat. Mach. Intell. 2, 573–584. 10.1038/s42256-020-00236-4

[B101] Keller K. Kirchgessner N. Khanna R. Siegwart R. Walter A. Aasen H. (2018). “Soybean leaf coverage estimation with machine learning and thresholding algorithms for field phenotyping,” in Proceedings of the British Machine Vision Conference (Newcastle: BMVA Press), 3–6.

[B102] Khemani R. G. Conti D. Alonzo T. A. Bart R. D. Newth C. J. (2009). Effect of tidal volume in children with acute hypoxemic respiratory failure. Intens. Care Med. 35, 1428–1437. 10.1007/s00134-009-1527-z 19533092

[B103] Kim B. Wattenberg M. Gilmer J. Cai C. Wexler J. Viegas F.. (2018). “Interpretability beyond feature attribution: quantitative testing with concept activation vectors (TCAV),” in International Conference on Machine Learning (PMLR), 2668–2677.

[B104] Kindermans P.-J. Hooker S. Adebayo J. Alber M. Schütt K. T. Dähne S.. (2019). “The (un) reliability of saliency methods,” in Explainable AI: Interpreting, Explaining and Visualizing Deep Learning (Switzerland: Springer), 267–280.

[B105] Kolhar S. Jagtap J. (2021). Plant trait estimation and classification studies in plant phenotyping using machine vision-a review. Inf. Process. Agric. 10, 114–135. 10.1016/j.inpa.2021.02.006

[B106] Krizhevsky A. Hinton G. (2009a). CIFAR-100 (Canadian Institute for Advanced Research) Dataset. Technical report, University of Toronto.

[B107] Krizhevsky A. Hinton G. (2009b). Learning Multiple Layers of Features From Tiny Images (TR-2009–R-208).

[B108] Krizhevsky A. Sutskever I. Hinton G. E. (2017). Imagenet classification with deep convolutional neural networks. Commun. ACM 60, 84–90. 10.1145/3065386

[B109] Kuleshov V. Fenner N. Ermon S. (2018). “Accurate uncertainties for deep learning using calibrated regression,” in International Conference on Machine Learning (Stockholm: ICML), 2796–2804.

[B110] Lapuschkin S. Binder A. Montavon G. Muller K.-R. Samek W. (2016). “Analyzing classifiers: fisher vectors and deep neural networks,” in Proceedings of the IEEE Conference on Computer Vision and Pattern Recognition (Las Vegas, NV), 2912–2920.

[B111] Larese M. G. Bayá A. E. Craviotto R. M. Arango M. R. Gallo C. Granitto P. M. (2014). Multiscale recognition of legume varieties based on leaf venation images. Exp. Syst. Appl. 41, 4638–4647. 10.1016/j.eswa.2014.01.029

[B112] Larsson S. Heintz F. (2020). Transparency in artificial intelligence. Int. Policy Rev. 9. 10.14763/2020.2.1469

[B113] LeCun Y. Boser B. Denker J. S. Henderson D. Howard R. E. Hubbard W.. (1989). Backpropagation applied to handwritten zip code recognition. Neural Comput. 1, 541–551. 10.1162/neco.1989.1.4.541

[B114] Lee H. Yune S. Mansouri M. Kim M. Tajmir S. H. Guerrier C. E.. (2019). An explainable deep-learning algorithm for the detection of acute intracranial haemorrhage from small datasets. Nat. Biomed. Eng. 3, 173–182. 10.1038/s41551-018-0324-9 30948806

[B115] Lee S. H. Goëau H. Bonnet P. Joly A. (2020). New perspectives on plant disease characterization based on deep learning. Comp. Electron. Agric. 170, 105220. 10.1016/j.compag.2020.10522035009139

[B116] Letham B. Rudin C. McCormick T. H. Madigan D. (2015). Interpretable classifiers using rules and bayesian analysis: building a better stroke prediction model. Ann. Appl. Stat. 9, 1350–1371. 10.1214/15-AOAS848

[B117] Li H. Xu Z. Taylor G. Studer C. Goldstein T. (2018). Visualizing the loss landscape of neural nets. Adv. Neural Inf. Process. Syst. (Montréal). 31.

[B118] Li L. Zhang Q. Huang D. (2014). A review of imaging techniques for plant phenotyping. Sensors 14, 20078–20111. 10.3390/s141120078 25347588 PMC4279472

[B119] Lin T.-Y. Goyal P. Girshick R. He K. Dollár P. (2017). “Focal loss for dense object detection,” in Proceedings of the IEEE International Conference on Computer Vision (Venice), 2980–2988.

[B120] Linardatos P. Papastefanopoulos V. Kotsiantis S. (2020). Explainable AI: a review of machine learning interpretability methods. Entropy 23, 18. 10.3390/e23010018 33375658 PMC7824368

[B121] Lipton Z. C. (2018). The mythos of model interpretability: in machine learning, the concept of interpretability is both important and slippery. Queue 16, 31–57. 10.1145/3236386.3241340

[B122] Liu L. Lu H. Li Y. Cao Z. (2020). High-throughput rice density estimation from transplantation to tillering stages using deep networks. Plant Phenom. 2020. 10.34133/2020/1375957 33313541 PMC7706318

[B123] Liu M. Shi J. Li Z. Li C. Zhu J. Liu S. (2016). Towards better analysis of deep convolutional neural networks. IEEE Trans. Vis. Comput. Graph. 23, 91–100. 10.1109/TVCG.2016.2598831 27576252

[B124] Liu S. Wang X. Liu M. Zhu J. (2017). Towards better analysis of machine learning models: a visual analytics perspective. Vis. Informat. 1, 48–56. 10.1016/j.visinf.2017.01.006

[B125] Long J. Shelhamer E. Darrell T. (2015). “Fully convolutional networks for semantic segmentation,” in Proceedings of the IEEE Conference on Computer Vision and Pattern Recognition (Boston, MA), 3431–3440. 10.1109/TPAMI.2016.2572683 27244717

[B126] Lou Y. Caruana R. Gehrke J. (2012). “Intelligible models for classification and regression,” in Proceedings of the 18th ACM SIGKDD International Conference on Knowledge Discovery and Data Mining (Beijing), 150–158.

[B127] Lu H. Cao Z. Xiao Y. Zhuang B. Shen C. (2017). TasselNet: counting maize tassels in the wild via local counts regression network. Plant Methods 13, 1–17. 10.1186/s13007-017-0224-0 29118821 PMC5664836

[B128] Lu H. Liu L. Li Y.-N. Zhao X.-M. Wang X.-Q. Cao Z.-G. (2021). TasselNetV3: explainable plant counting with guided upsampling and background suppression. IEEE Transact. Geosci. Remote Sens. 60, 1–15. 10.1109/TGRS.2021.3058962

[B129] Lube V. Noyan M. A. Przybysz A. Salama K. Blilou I. (2022). MultipleXLab: a high-throughput portable live-imaging root phenotyping platform using deep learning and computer vision. Plant Methods 18, 38. 10.1186/s13007-022-00864-4 35346267 PMC8958799

[B130] Lundberg S. M. Lee S.-I. (2017). A unified approach to interpreting model predictions. Adv. Neural Inf. Process. Syst. (Long Beach, CA) 30.

[B131] Lyons J. B. (2013). “Being transparent about transparency: a model for human-robot interaction,” in 2013 AAAI Spring Symposium Series (Palo Alto, CA).

[B132] Madec S. Jin X. Lu H. De Solan B. Liu S. Duyme F.. (2019). Ear density estimation from high resolution RGB imagery using deep learning technique. Agric. For. Meteorol. 264, 225–234. 10.1016/j.agrformet.2018.10.013

[B133] Maghiaoui A. Bouguyon E. Cuesta C. Perrine-Walker F. Alcon C. Krouk G.. (2020). The arabidopsis NRT1. 1 transceptor coordinately controls auxin biosynthesis and transport to regulate root branching in response to nitrate. J. Exp. Bot. 71, 4480–4494. 10.1093/jxb/eraa242 32428238

[B134] Mairhofer S. Zappala S. Tracy S. Sturrock C. Bennett M. J. Mooney S. J.. (2013). Recovering complete plant root system architectures from soil via x-ray μ-computed tomography. Plant Methods 9, 1–7. 10.1186/1746-4811-9-8 23514198 PMC3615952

[B135] Mardanisamani S. Maleki F. Hosseinzadeh Kassani S. Rajapaksa S. Duddu H. Wang M.. (2019). “Crop lodging prediction from uav-acquired images of wheat and canola using a dcnn augmented with handcrafted texture features,” in Proceedings of the IEEE/CVF Conference on Computer Vision and Pattern Recognition Workshops (Long Beach, CA).

[B136] Mehdiyev N. Fettke P. (2021). “Explainable artificial intelligence for process mining: a general overview and application of a novel local explanation approach for predictive process monitoring,” in Interpretable artificial intelligence: a perspective of granular computing. Studies in computational intelligence, Vol. 937, eds W. Pedrycz and S. M. Chen (Cham: Springer). 10.1007/978-3-030-64949-4_1

[B137] Miller T. (2019). Explanation in artificial intelligence: insights from the social sciences. Artif. Intell. 267, 1–38. 10.1016/j.artint.2018.07.007

[B138] Minamikawa M. F. Nonaka K. Hamada H. Shimizu T. Iwata H. (2022). Dissecting breeders' sense via explainable machine learning approach: application to fruit peelability and hardness in citrus. Front. Plant Sci. 13, 832749. 10.3389/fpls.2022.832749 35222489 PMC8867066

[B139] Mohanty S. P. Hughes D. P. Salathé M. (2016). Using deep learning for image-based plant disease detection. Front. Plant Sci. 7, 1419. 10.3389/fpls.2016.01419 27713752 PMC5032846

[B140] Mohseni S. Block J. E. Ragan E. D. (2018). A human-grounded evaluation benchmark for local explanations of machine learning. arXiv:1801.05075v2.

[B141] Mohseni S. Zarei N. Ragan E. D. (2021). A multidisciplinary survey and framework for design and evaluation of explainable ai systems. ACM Transact. Interact. Intell. Syst. 11, 1–45. 10.1145/3387166

[B142] Moore J. D. Swartout W. R. (1988). Explanation in Expert Systems: A Survey. Technical report, University of Southern California Marina Del Rey Information Sciences Inst, (Los Angeles, CA).

[B143] Mortensen A. K. Skovsen S. Karstoft H. Gislum R. (2019). “The oil radish growth dataset for semantic segmentation and yield estimation,” in 2019 IEEE/CVF Conference on Computer Vision and Pattern Recognition Workshops (CVPRW) (Long Beach, CA: IEEE), 2703–2710.

[B144] Mostafa S. Mondal D. Beck M. Bidinosti C. Henry C. Stavness I. (2021). “Visualizing feature maps for model selection in convolutional neural networks,” in Proceedings of the IEEE/CVF International Conference on Computer Vision (Montreal), 1362–1371.

[B145] Mostafa S. Mondal D. Beck M. A. Bidinosti C. P. Henry C. J. Stavness I. (2022). Leveraging guided backpropagation to select convolutional neural networks for plant classification. Front. Artif. Intell. 5, 871162. 10.3389/frai.2022.871162 35647528 PMC9132261

[B146] Mundermann L. Erasmus Y. Lane B. Coen E. Prusinkiewicz P. (2005). Quantitative modeling of arabidopsis development. Plant Physiol. 139, 960–968. 10.1104/pp.105.060483 16183852 PMC1256009

[B147] Nagasubramanian K. Jones S. Singh A. K. Sarkar S. Singh A. Ganapathysubramanian B. (2019). Plant disease identification using explainable 3D deep learning on hyperspectral images. Plant Methods 15, 1–10. 10.1186/s13007-019-0479-8 31452674 PMC6702735

[B148] Nagasubramanian K. Singh A. K. Singh A. Sarkar S. Ganapathysubramanian B. (2020). Usefulness of interpretability methods to explain deep learning based plant stress phenotyping. arXiv:2007.05729v1.

[B149] Nageshrao S. Tseng H. E. Filev D. (2019). “Autonomous highway driving using deep reinforcement learning,” in 2019 IEEE International Conference on Systems, Man and Cybernetics (SMC) (Italy: IEEE), 2326–2331.

[B150] Nakhle F. Harfouche A. L. (2021). Ready, steady, go AI: a practical tutorial on fundamentals of artificial intelligence and its applications in phenomics image analysis. Patterns 2, 100323. 10.1016/j.patter.2021.100323 34553170 PMC8441561

[B151] Nazki H. Yoon S. Fuentes A. Park D. S. (2020). Unsupervised image translation using adversarial networks for improved plant disease recognition. Comp. Electron. Agric. 168, 105117. 10.1016/j.compag.2019.105117

[B152] Newell A. Yang K. Deng J. (2016). “Stacked hourglass networks for human pose estimation,” in European Conference on Computer Vision (Amsterdam: Springer), 483–499.

[B153] Ngugi L. C. Abelwahab M. Abo-Zahhad M. (2021). Recent advances in image processing techniques for automated leaf pest and disease recognition-a review. Inf. Process. Agric. 8, 27–51. 10.1016/j.inpa.2020.04.004

[B154] Nguyen A. Dosovitskiy A. Yosinski J. Brox T. Clune J. (2016). Synthesizing the preferred inputs for neurons in neural networks via deep generator networks. Adv. Neural Inf. Process. Syst. (Barcelona), 29, 3387–3395.

[B155] Nigri E. Ziviani N. Cappabianco F. Antunes A. Veloso A. (2020). “Explainable deep cnns for mri-based diagnosis of Alzheimer's disease,” in 2020 International Joint Conference on Neural Networks (IJCNN) (Glasgow: IEEE), 1–8.

[B156] Oliveira M. F. Nelson R. L. Geraldi I. O. Cruz C. D. de Toledo J. F. F. (2010). Establishing a soybean germplasm core collection. Field Crops Res. 119, 277–289. 10.1016/j.fcr.2010.07.021

[B157] Pound M. P. Atkinson J. A. Wells D. M. Pridmore T. P. French A. P. (2017). “Deep learning for multi-task plant phenotyping,” in Proceedings of the IEEE International Conference on Computer Vision Workshops (Venice), 2055–2063.

[B158] Preece A. (2018). Asking “why” in AI: explainability of intelligent systems-perspectives and challenges. Intell. Syst. Account. Finan. Manag. 25, 63–72. 10.1002/isaf.1422

[B159] ProPublica (2016). COMPAS data and analysis for ‘Machine Bias'. https://github.com/propublica/compas-analysis

[B160] Puyol-Antón E. Ruijsink B. Piechnik S. K. Neubauer S. Petersen S. E. Razavi R.. (2021). “Fairness in cardiac mr image analysis: an investigation of bias due to data imbalance in deep learning based segmentation,” in International Conference on Medical Image Computing and Computer-Assisted Intervention (Springer), 413–423.

[B161] Raaijmakers S. (2019). Artificial intelligence for law enforcement: challenges and opportunities. IEEE Sec. Privacy 17, 74–77. 10.1109/MSEC.2019.292564937434229

[B162] Radford A. Metz L. Chintala S. (2015). Unsupervised representation learning with deep convolutional generative adversarial networks. arXiv. arXiv:1511.06434v2.

[B163] Rao Q. Frtunikj J. (2018). “Deep learning for self-driving cars: chances and challenges,” in Proceedings of the 1st International Workshop on Software Engineering for AI in Autonomous Systems (Gothenburg), 35–38.

[B164] Reinelt L. Whitaker J. Kazakou E. Bonnal L. Bastianelli D. Bullock J. M.. (2023). Drought effects on root and shoot traits and their decomposability. Funct. Ecol. 37, 1044–1054. 10.1111/1365-2435.14261

[B165] Remans T. Nacry P. Pervent M. Filleur S. Diatloff E. Mounier E.. (2006). The arabidopsis NRT1. 1 transporter participates in the signaling pathway triggering root colonization of nitrate-rich patches. Proc. Natl. Acad. Sci. U. S. A. 103, 19206–19211. 10.1073/pnas.0605275103 17148611 PMC1748200

[B166] Ren C. Kim D.-K. Jeong D. (2020). A survey of deep learning in agriculture: techniques and their applications. J. Inf. Process. Syst. 16, 1015–1033. 10.3745/JIPS.04.0187

[B167] Ren M. Zemel R. S. (2017). “End-to-end instance segmentation with recurrent attention,” in Proceedings of the IEEE Conference on Computer Vision and Pattern Recognition (Honolulu, HI), 6656–6664.

[B168] Research (2020). Deep learning: Global Markets. Available online at: https://www.asdreports.com/market-research-report-567969/deep-learning-global-markets (accessed January, 2023)

[B169] Rezaei M. Terauchi M. (2013). “Vehicle detection based on multi-feature clues and dempster-shafer fusion theory,” in Pacific-Rim Symposium on Image and Video Technology (Springer), 60–72.

[B170] Ribeiro M. T. Singh S. Guestrin C. (2016). ““Why should i trust you?” explaining the predictions of any classifier,” in *Proceedings of the 22nd ACM SIGKDD International Conference on Knowledge Discovery and Data Mining* (San Francisco, CA), 1135–1144.

[B171] Ribeiro M. T. Singh S. Guestrin C. (2018). “Anchors: high-precision model-agnostic explanations,” in Proceedings of the AAAI Conference on Artificial Intelligence, Vol. 32 (New Orleans, LA).

[B172] Russakovsky O. Deng J. Su H. Krause J. Satheesh S. Ma S.. (2015). Imagenet large scale visual recognition challenge. Int. J. Comput. Vis. 115, 211–252. 10.1007/s11263-015-0816-y

[B173] Saeed M. Villarroel M. Reisner A. T. Clifford G. Lehman L.-W. Moody G.. (2011). Multiparameter intelligent monitoring in intensive care II (MIMIC-II): a public-access intensive care unit database. Crit. Care Med. 39, 952. 10.1097/CCM.0b013e31820a92c6 21283005 PMC3124312

[B174] Samek W. Binder A. Montavon G. Lapuschkin S. Müller K.-R. (2016). Evaluating the visualization of what a deep neural network has learned. IEEE Transact. Neural Netw. Learn. Syst. 28, 2660–2673. 10.1109/TNNLS.2016.2599820 27576267

[B175] Samek W. Müller K.-R. (2019). “Towards explainable artificial intelligence,” in Explainable AI: Interpreting, Explaining and Visualizing Deep Learning (Switzerland: Springer), 5–22.

[B176] Sarker I. H. (2021). Deep learning: a comprehensive overview on techniques, taxonomy, applications and research directions. SN Comp. Sci. 2, 420. 10.1007/s42979-021-00815-1 34426802 PMC8372231

[B177] Schetinin V. Fieldsend J. E. Partridge D. Coats T. J. Krzanowski W. J. Everson R. M.. (2007). Confident interpretation of bayesian decision tree ensembles for clinical applications. IEEE Transact. Inf. Technol. Biomed. 11, 312–319. 10.1109/TITB.2006.880553 17521081

[B178] Schramowski P. Stammer W. Teso S. Brugger A. Herbert F. Shao X.. (2020). Making deep neural networks right for the right scientific reasons by interacting with their explanations. Nat. Mach. Intell. 2, 476–486. 10.1038/s42256-020-0212-3

[B179] Seidenthal K. Panjvani K. Chandnani R. Kochian L. Eramian M. (2022). Iterative image segmentation of plant roots for high-throughput phenotyping. Sci. Rep. 12, 16563. 10.1038/s41598-022-19754-9 36195610 PMC9532414

[B180] Selvaraju R. R. Cogswell M. Das A. Vedantam R. Parikh D. Batra D. (2017). “Grad-CAM: visual explanations from deep networks via gradient-based localization,” in Proceedings of the IEEE International Conference on Computer Vision (Venice), 618–626.

[B181] Shen C. Liu L. Zhu L. Kang J. Wang N. Shao L. (2020). High-throughput *in situ* root image segmentation based on the improved DeepLabv3+ method. Front. Plant Sci. 11, 576791. 10.3389/fpls.2020.576791 33193519 PMC7604297

[B182] Simonyan K. Vedaldi A. Zisserman A. (2013). Deep inside convolutional networks: visualising image classification models and saliency maps. arXiv:1312.6034v2.

[B183] Singh A. K. Ganapathysubramanian B. Sarkar S. Singh A. (2018). Deep learning for plant stress phenotyping: trends and future perspectives. Trends Plant Sci. 23, 883–898. 10.1016/j.tplants.2018.07.004 30104148

[B184] Skovsen S. Dyrmann M. Mortensen A. K. Laursen M. S. Gislum R. Eriksen J.. (2019). “The grassclover image dataset for semantic and hierarchical species understanding in agriculture,” in Proceedings of the IEEE/CVF Conference on Computer Vision and Pattern Recognition Workshops (Long Beach, CA).

[B185] Smilkov D. Thorat N. Kim B. Viégas F. Wattenberg M. (2017). Smoothgrad: removing noise by adding noise. arXiv:1706.03825v1.

[B186] Soares E. Angelov P. P. Costa B. Castro M. P. G. Nageshrao S. Filev D. (2020). Explaining deep learning models through rule-based approximation and visualization. IEEE Transact. Fuzzy Syst. 29, 2399–2407. 10.1109/TFUZZ.2020.2999776

[B187] Soille P. (1999). Morphological Image Analysis: Principles and Applications, Vol. 2. Berlin; Heidelberg: Springer.

[B188] Song Q. Hyten D. L. Jia G. Quigley C. V. Fickus E. W. Nelson R. L.. (2013). Development and evaluation of soysnp50k, a high-density genotyping array for soybean. PLoS ONE 8, e54985. 10.1371/journal.pone.0054985 23372807 PMC3555945

[B189] Springenberg J. T. Dosovitskiy A. Brox T. Riedmiller M. (2014). Striving for simplicity: The all convolutional net. arXiv:1412.6806v3.

[B190] Sünderhauf N. Brock O. Scheirer W. Hadsell R. Fox D. Leitner J.. (2018). The limits and potentials of deep learning for robotics. Int. J. Rob. Res. 37, 405–420. 10.1177/0278364918770733

[B191] Swartout W. R. (1983). Xplain: a system for creating and explaining expert consulting programs. Artif. Intell. 21, 285–325. 10.1016/S0004-3702(83)80014-9

[B192] Szegedy C. Liu W. Jia Y. Sermanet P. Reed S. Anguelov D.. (2015). “Going deeper with convolutions,” in Proceedings of the IEEE Conference on Computer Vision and Pattern Recognition (Boston, MA), 1–9.

[B193] Szegedy C. Vanhoucke V. Ioffe S. Shlens J. Wojna Z. (2016). “Rethinking the inception architecture for computer vision,” in Proceedings of the IEEE Conference on Computer Vision and Pattern Recognition (Las Vegas, LA), 2818–2826.

[B194] Taghavi Namin S. Esmaeilzadeh M. Najafi M. Brown T. B. Borevitz J. O. (2018). Deep phenotyping: deep learning for temporal phenotype/genotype classification. Plant Methods 14, 1–14. 10.1186/s13007-018-0333-4 30087695 PMC6076396

[B195] Tan S. Caruana R. Hooker G. Lou Y. (2018). “Distill-and-compare: auditing black-box models using transparent model distillation,” in Proceedings of the 2018 AAAI/ACM Conference on AI, Ethics, and Society (New Orleans, LA), 303–310.

[B196] Thesma V. Mohammadpour Velni J. (2022). Plant root phenotyping using deep conditional gans and binary semantic segmentation. Sensors 23, 309. 10.3390/s23010309 36616905 PMC9823511

[B197] Thomas S. M. Lefevre J. G. Baxter G. Hamilton N. A. (2021). Interpretable deep learning systems for multi-class segmentation and classification of non-melanoma skin cancer. Med. Image Anal. 68, 101915. 10.1016/j.media.2020.101915 33260112

[B198] Tian H. Zhu T. Liu W. Zhou W. (2022). Image fairness in deep learning: problems, models, and challenges. Neur. Comp. Appl. 34, 12875–12893. 10.1007/s00521-022-07136-137203610

[B199] Tjoa E. Guan C. (2020). A survey on explainable artificial intelligence (XAI): toward medical xai. IEEE Transact. Neural Netw. Learn. Syst. 32, 4793–4813. 10.1109/TNNLS.2020.3027314 33079674

[B200] Toda Y. Okura F. (2019). How convolutional neural networks diagnose plant disease. Plant Phenom. 2019. 10.34133/2019/9237136 33313540 PMC7706313

[B201] Tonekaboni S. Joshi S. McCradden M. D. Goldenberg A. (2019). “What clinicians want: contextualizing explainable machine learning for clinical end use,” in Machine Learning for Healthcare Conference (PMLR), 59–380.

[B202] Tripathi A. D. Mishra R. Maurya K. K. Singh R. B. Wilson D. W. (2019). “Estimates for world population and global food availability for global health,” in The Role of Functional Food Security in Global Health (Amsterdam: Elsevier), 3–24.

[B203] Tsaftaris A. S. Scharr H. (2017). Leaf Segmentation and Counting Challenges. Available online at: https://www.plant-phenotyping.org/CVPPP2017-challenge (accessed March 16, 2023).

[B204] Ubbens J. Cieslak M. Prusinkiewicz P. Parkin I. Ebersbach J. Stavness I. (2020). Latent space phenotyping: automatic image-based phenotyping for treatment studies. Plant Phenom. 2020. 10.34133/2020/5801869 33313558 PMC7706325

[B205] Ubbens J. Cieslak M. Prusinkiewicz P. Stavness I. (2018). The use of plant models in deep learning: an application to leaf counting in rosette plants. Plant Methods 14, 1–10. 10.1186/s13007-018-0273-z 29375647 PMC5773030

[B206] Ubbens J. R. Stavness I. (2017). Deep plant phenomics: a deep learning platform for complex plant phenotyping tasks. Front. Plant Sci. 8, 1190. 10.3389/fpls.2017.01190 28736569 PMC5500639

[B207] Uchiyama H. Sakurai S. Mishima M. Arita D. Okayasu T. Shimada A.. (2017). “An easy-to-setup 3d phenotyping platform for komatsuna dataset,” in Proceedings of the IEEE International Conference on Computer Vision Workshops (Venice), 2038–2045.

[B208] Valerio Giuffrida M. Scharr H. Tsaftaris S. A. (2017). “Arigan: synthetic arabidopsis plants using generative adversarial network,” in Proceedings of the IEEE International Conference on Computer Vision Workshops (Venice), 2064–2071.

[B209] Van Lent M. Fisher W. Mancuso M. (2004). “An explainable artificial intelligence system for small-unit tactical behavior,” in Proceedings of the National Conference on Artificial Intelligence (Menlo Park, CA; Cambridge, MA; London: AAAI Press; MIT Press; 1999), 900–907.

[B210] Vaswani A. Shazeer N. Parmar N. Uszkoreit J. Jones L. Gomez A. N.. (2017). Attention is all you need. Adv. Neural Inf. Process. Syst. (Long Beach, CA), 30.

[B211] Veley K. M. Berry J. C. Fentress S. J. Schachtman D. P. Baxter I. Bart R. (2017). High-throughput profiling and analysis of plant responses over time to abiotic stress. Plant Direct 1, e00023. 10.1002/pld3.23 31245669 PMC6508565

[B212] Vilone G. Longo L. (2020). Explainable artificial intelligence: a systematic review. arXiv.

[B213] Vilone G. Longo L. (2021). Notions of explainability and evaluation approaches for explainable artificial intelligence. Inf. Fus. 76, 89–106. 10.1016/j.inffus.2021.05.00934844219

[B214] Vit A. Shani G. Bar-Hillel A. (2019). “Length phenotyping with interest point detection,” in Proceedings of the IEEE/CVF Conference on Computer Vision and Pattern Recognition Workshops (Long Beach, CA).

[B215] Wah C. Branson S. Welinder P. Perona P. Belongie S. (2011). The Caltech-UCSD Birds-200-2011 Dataset. Tech. Rep. CNS-TR-2011-001, California Institute of Technology.

[B216] Wang T.-C. Liu M.-Y. Zhu J.-Y. Tao A. Kautz J. Catanzaro B. (2018). “High-resolution image synthesis and semantic manipulation with conditional gans,” in Proceedings of the IEEE Conference on Computer Vision and Pattern Recognition (Salt Lake City, UT), 8798–8807.

[B217] Wang X. Peng Y. Lu L. Lu Z. Bagheri M. Summers R. M. (2017). “Chestx-ray8: hospital-scale chest x-ray database and benchmarks on weakly-supervised classification and localization of common thorax diseases,” in Proceedings of the IEEE Conference on Computer Vision and Pattern Recognition (Honolulu, HI), 2097–2106.

[B218] Wang Y.-Y. Cheng Y.-H. Chen K.-E. Tsay Y.-F. (2018). Nitrate transport, signaling, and use efficiency. Annu. Rev. Plant Biol. 69, 85–122. 10.1146/annurev-arplant-042817-040056 29570365

[B219] Weerts H. J. van Ipenburg W. Pechenizkiy M. (2019). A human-grounded evaluation of shap for alert processing. arXiv:1907.03324v1.

[B220] Wei K. Chen B. Zhang J. Fan S. Wu K. Liu G.. (2022). Explainable deep learning study for leaf disease classification. Agronomy 12, 1035. 10.3390/agronomy12051035

[B221] Weiner M. W. Veitch D. P. Aisen P. S. Beckett L. A. Cairns N. J. Green R. C.. (2013). The Alzheimer's disease neuroimaging initiative: a review of papers published since its inception. Alzheimers Dement. 9, e111–e194. 10.1016/j.jalz.2013.05.1769 23932184 PMC4108198

[B222] Wilson M. H. Holman T. J. Sørensen I. Cancho-Sanchez E. Wells D. M. Swarup R.. (2015). Multi-omics analysis identifies genes mediating the extension of cell walls in the arabidopsis thaliana root elongation zone. Front. Cell Dev. Biol. 3, 10. 10.3389/fcell.2015.00010 25750913 PMC4335395

[B223] Wolfert S. Ge L. Verdouw C. Bogaardt M.-J. (2017). Big data in smart farming-a review. Agric. Syst. 153, 69–80. 10.1016/j.agsy.2017.01.023

[B224] Xie X. Niu J. Liu X. Chen Z. Tang S. Yu S. (2021). A survey on incorporating domain knowledge into deep learning for medical image analysis. Med. Image Anal. 69, 101985. 10.1016/j.media.2021.101985 33588117

[B225] Xu F. Uszkoreit H. Du Y. Fan W. Zhao D. Zhu J. (2019). “Explainable AI: a brief survey on history, research areas, approaches and challenges,” in CCF International Conference on Natural Language Processing and Chinese Computing (Dunhuang: Springer), 563–574.

[B226] Xu Z. York L. M. Seethepalli A. Bucciarelli B. Cheng H. Samac D. A. (2022). Objective phenotyping of root system architecture using image augmentation and machine learning in alfalfa (medicago sativa l.). Plant Phenom. 2022. 10.34133/2022/9879610 35479182 PMC9012978

[B227] Yang M. Kim B. (2019). Benchmarking attribution methods with relative feature importance. arXiv:1907.09701v2.

[B228] Yang X. He X. Zhao J. Zhang Y. Zhang S. Xie P. (2020). Covid-CT-dataset: a CT scan dataset about Covid-19. arXiv:2003.13865v3.

[B229] Yasrab R. Atkinson J. A. Wells D. M. French A. P. Pridmore T. P. Pound M. P. (2019). Rootnav 2.0: deep learning for automatic navigation of complex plant root architectures. GigaScience, 8, giz123. 10.1093/gigascience/giz123 31702012 PMC6839032

[B230] Yasrab R. Zhang J. Smyth P. Pound M. P. (2021). Predicting plant growth from time-series data using deep learning. Remote Sens. 13, 331. 10.3390/rs13030331

[B231] Zeiler M. D. Fergus R. (2014). “Visualizing and understanding convolutional networks,” in European Conference on Computer Vision (Zurich: Springer), 818–833.

[B232] Zhou B. Khosla A. Lapedriza A. Oliva A. Torralba A. (2016). “Learning deep features for discriminative localization,” in Proceedings of the IEEE Conference on Computer Vision and Pattern Recognition (Las Vegas, NV), 2921–2929.

[B233] Zhu J.-Y. Park T. Isola P. Efros A. A. (2017). “Unpaired image-to-image translation using cycle-consistent adversarial networks,” in Proceedings of the IEEE International Conference on Computer Vision (Venice), 2223–2232.

[B234] Zintgraf L. M. Cohen T. S. Adel T. Welling M. (2017). Visualizing deep neural network decisions: Prediction difference analysis. arXiv:1702.04595v1.

